# Ligilactobacillus Murinus and Lactobacillus Johnsonii Suppress Macrophage Pyroptosis in Atherosclerosis through Butyrate‐GPR109A‐GSDMD Axis

**DOI:** 10.1002/advs.202501707

**Published:** 2025-07-29

**Authors:** Rui Hua, Ning Ding, Yiming Hua, Xiaoke Wang, Yu Xu, Xiangrui Qiao, Xue Shi, Ting Bai, Ying Xiong, Xiaozhen Zhuo, Chong Fan, Juan Zhou, Yue Wu, Junhui Liu, Zuyi Yuan, Ting Li

**Affiliations:** ^1^ Department of Cardiovascular Medicine First Affiliated Hospital Xi'an Jiaotong University Xi'an Shaanxi 710061 China; ^2^ Key Laboratory of Molecular Cardiology Shaanxi Province Xi'an Shaanxi 710061 China; ^3^ Key Laboratory of Environment and Genes Related to Diseases Ministry of Education Xi'an Shaanxi 710061 China; ^4^ Department of Clinical Laboratory First Affiliated Hospital Xi'an Jiaotong University Xi'an Shaanxi 710061 China; ^5^ Biobank First Affiliated Hospital Xi'an Jiaotong University Xi'an Shaanxi 710061 China

**Keywords:** atherosclerosis, butyrate, lactobacillus johnsonii, ligilactobacillus murinus, pyroptosis

## Abstract

Gut microbiota and their metabolites are remarkable regulators in atherosclerosis. Oral drugs such as aspirin have recently been found to modulate the gut microbiome. However, the roles of drug‐microbiota‐metabolite interactions in atherosclerosis have not been explored. Herein, two gut probiotics, *Ligilactobacillus murinus* (*L. murinus*) and *Lactobacillus johnsonii* (*L. johnsonii*), are identified from mouse models and human cohorts, which are positively correlated with aspirin usage. Specifically, the eradication of these two species eliminated aspirin's anti‐atherosclerotic effects, while their transplantation exhibited therapeutic effects against atherosclerosis. Integrative analysis of metagenomic and metabolomic data showed that elevated levels of butyrate are associated with these two species. Mechanically, *L. murinus* and *L. johnsonii* form symbiotic networks with butyrate‐producing bacteria such as *Allobaculum*. This study confirmed that gut microbes produce butyrate, which helps preserve the gut barrier and prevents the leakage of lipopolysaccharides. By integrating molecular biology and single‐cell sequencing data, G protein‐coupled receptor 109A (GPR109A) is confirmed as the direct target of butyrate. Through the activation of GPR109A, butyrate produced by *L. murinus* and *L. johnsonii* suppressed the expression of Gasdermin D (GSDMD) in the pyroptosis of macrophages during atherosclerosis. These findings offer novel insights into the drug‐microbiota axis that can be targeted to improve the treatment of atherosclerosis.

## Graphical abstract

Oral aspirin administration induces intestinal expansion of *Lactobacillus murinus* and *Lactobacillus johnsonii*, which suppresses the progression of atherosclerosis. This microbial expansion significantly enhances butyrate production by providing lactate as a metabolic substrate, thereby fostering the growth of butyrate‐producing bacteria. Butyrate plays a pivotal role in maintaining gut barrier integrity, preventing the translocation of lipopolysaccharide (LPS) into systemic circulation. Mechanistically, gut‐derived butyrate binds to the G‐protein‐coupled receptor GPR109A on macrophages, inhibiting LPS‐induced pyroptosis via suppression of the GSDMD/NLRP3/IL‐1β signaling pathway. This attenuation of inflammatory responses leads to reduced plaque inflammation and ameliorated atherosclerosis progression.

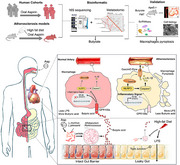

## 1 Introduction

Atherosclerosis is a chronic and progressive disease featuring the highest morbidity and mortality rates worldwide.^[^ ^1^ ^]^ During the past 30 years, aspirin has been considered the cornerstone in the primary and secondary prevention of atherosclerotic cardiovascular disease (ASCVD).^[^ ^2^ ^]^

The best‐characterized molecular mechanism of action of low‐dose aspirin in preventing atherothrombosis is related to the irreversible nature of platelet cyclooxygenase‐1 (COX‐1) inactivation.^[^ ^2^, ^3^ ^]^ Nevertheless, the COX‐independent effects of aspirin cannot be ignored, since the anti‐inflammatory effects of aspirin were also confirmed in COX‐1knockout mice.^[^ ^4^ ^]^ In multiple animal models, low‐dose aspirin was found to suppress vascular inflammation and increase the stability of atherosclerotic plaques, which contributes to its anti‐atherogenic effect.^[^ ^5^ ^]^ More recently, we reported that aspirin interacts gut microbiome, which mediates its damage to intestinal homeostasis.^[^ ^6^ ^]^ We found that aspirin shifted the gut microbial composition and metabolite spectrum. The decreased abundance of *Parabacteroides* has been focused on, as well as the subsequent regulation of secondary bile acids in the intestinal injury model. Interestingly, we also observed an increase in several species, including *Lactobacillus*.^[^ ^6^ ^]^ However, the impact of aspirin on the gut microbial community in an atherosclerotic model and its functional metabolites has not been fully explored.

The gut bacterial community plays an important role in regulating host physiology and pathology, particularly in the initiation and progression of atherosclerosis.^[^ ^7^ ^]^ Since the discovery of the relationship between gut flora‐dependent metabolism of dietary phosphatidylcholine and cardiovascular disease (CVD) pathogenesis,^[^ ^8^ ^]^ a variety of gut microbial metabolites have been proven functional in modulating the progression of atherosclerosis.^[^ ^9^ ^]^ These metabolites include short‐chain fatty acids (SCFAs), secondary bile acids, trimethylamine N‐oxide (TMAO), and phenylacetylglutamine. In particular, accumulating evidence has shown that gut microbiota influences atherosclerosis by SCFAs in numerous mechanistic animal model studies.^[^ ^9^, ^10^ ^]^ It has been reported that SCFAs play pivotal roles in regulating the activation, infiltration, and polarization of macrophages during the progression of atherosclerosis.^[^ ^11^ ^]^ However, the specific signaling pathways underlying the regulation of SCFAs on intraplaque macrophages remain obscure.

Atherosclerosis is a progressive inflammatory disorder of the arterial vessel wall, characterized by substantial infiltration of macrophages.^[^ ^1^, ^12^ ^]^ Macrophages, coupled with their plasticity, are hallmarks of ASCVD, contributing to the progression of plaques and the stabilization of existing atherosclerosis.^[^ ^13^ ^]^ Intraplaque macrophages have been observed to undergo programmed cell death, which plays a crucial role in the formation and expansion of the necrotic core during plaque progression. Pyroptosis, a form of programmed cell death, is characterized by the formation of plasma membrane pores through the gasdermin protein family and the release of pro‐inflammatory cytokines.^[^ ^14^ ^]^ In patients with atherosclerosis, elevated levels of GSDMD mRNA have been observed in peripheral blood monocytes, suggesting that GSDMD may serve as a biomarker for pyroptosis in atherosclerotic plaques.^[^ ^15^ ^]^ Recent studies conducted by our group and others have also shown that macrophage pyroptosis is involved in the initiation, progression, and complications of atherosclerosis.^[^ ^16^ ^]^ A more recent study suggests an interaction between gut microbial metabolites and macrophage pyroptosis.^[^ ^17^ ^]^ However, the role of the gut microbiome in regulating macrophage function and its subsequent impact on the progression of atherosclerosis has not been fully elucidated.

In this study, we identified two probiotics, *L. murinus* and *L. johnsonii*, that exhibit anti‐atherosclerotic effects in both human cohorts and animal models. Our findings indicate that a high‐fat diet (HFD) disrupts the gut microbiota and compromises the gut barrier; however, these adverse effects can be mitigated through aspirin treatment and the gut colonization by these two probiotics. Notably, the gut microbial metabolite butyric acid was found to be enhanced by these probiotics, which helps preserve the gut barrier and rescue HFD‐induced atherosclerosis by inhibiting intraplaque macrophage pyroptosis. This novel probiotics‐SCFA‐GPR109A‐pyroptosis axis suppresses downstream inflammatory pathways, thereby inducing therapeutic effects for the prevention of atherosclerosis.

## 2 Results

### 2.1 *L. Murinus* and *L. Johnsonii* are Modulated by Aspirin in HFD‐Treated Mice

**Figure 1:**
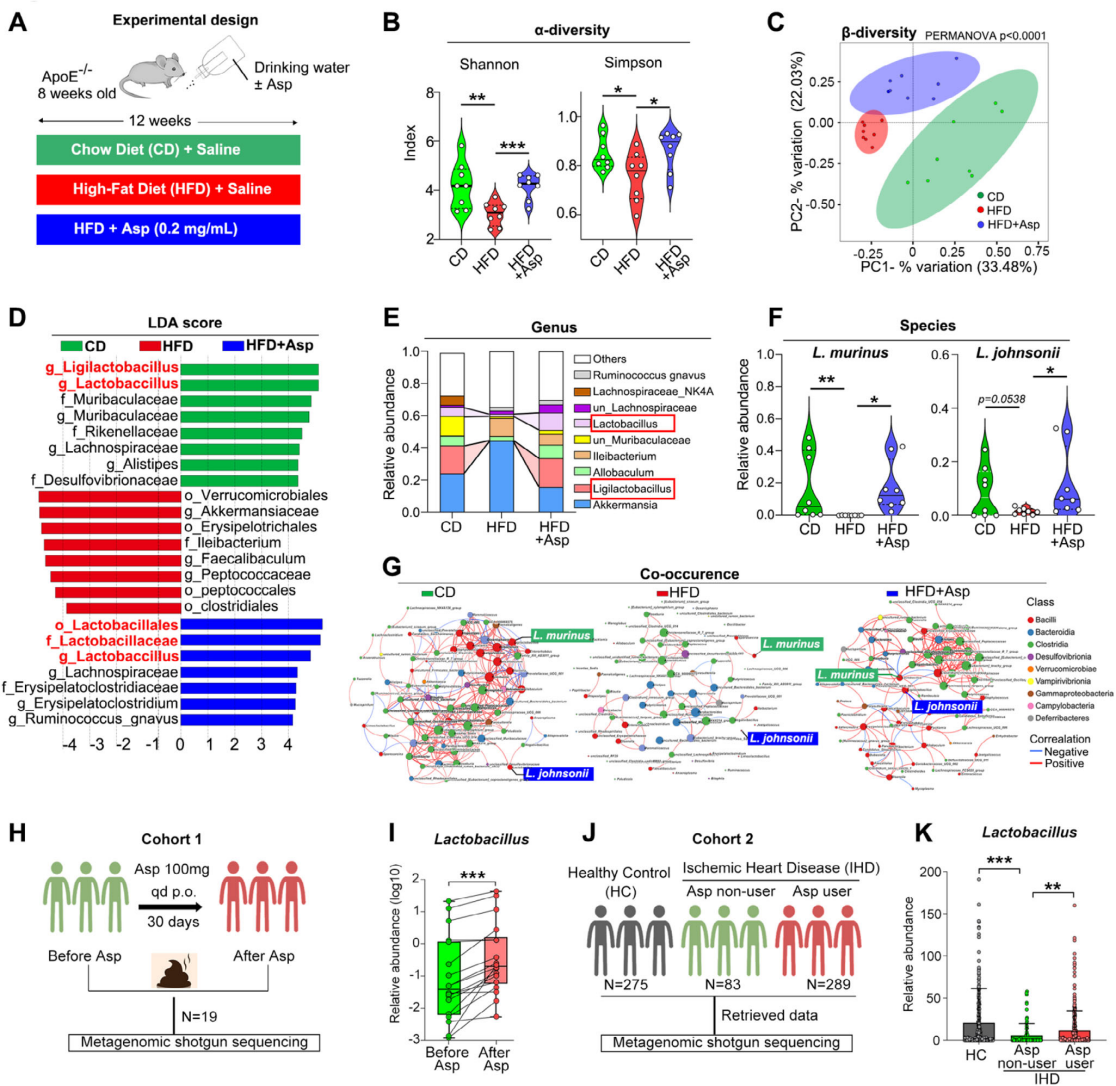
*L. murinus* and *L. johnsonii* are modulated by aspirin in HFD‐induced mouse model. A) The experimental design shows that the CD‐ and HFD‐fed *ApoE* ^−/−^ mice were treated with low‐dose aspirin (0.2 mg mL^−1^ in their drinking water). B) Changes in α‐diversity (Shannon and Simpson indexes) of the gut microbiome in the three groups. n = 8/group. C) Principal co‐ordinates analysis (PCoA) based on the β‐diversity indexes (bray‐curtis distances) in the three groups. n = 8/group. D) Linear discriminant analysis effect size (LEfSe) analysis of relative abundance in the microbiome in the three groups. E,F) Changes of relative abundance of gut microbiota at levels of genus (E) and species among the three groups. G) Co‐occurrence network of gut microbes in the three groups. Connecting lines indicate the absolute values of Spearman's rank correlation coefficient >0.30. Core genus data (top 80 abundant genus) were extracted, and the colours of nodes indicated different classes. Red links, positive correlation; Blue links, negative correlation. H) For Cohort 1, 19 healthy volunteers were treated with aspirin for 30 days. Whole‐genome shotgun sequencing and metabolomic detection of stool samples were conducted before and after aspirin treatment. I) Relative abundance of *Lactobacillus* at genus level in fecal samples in Cohort 1. J) For Cohort 2, the metabolomic and metagenomic data were retrieved from a published study^[^ ^46^ ^]^ enrolling 647 patients with cardiometabolic disease. The patients were divided into 3 groups by medical history: HC volunteers (N = 275), patients with IHD treated with (N = 289) or without aspirin (N = 83). K) Relative abundance of the *Lactobacillus* genus in fecal samples in Cohort 2. Data are expressed as mean ± s.d. One‐way ANOVA followed by Fisher's LSD post hoc test. (B,F,K) Two‐tailed wilcoxon rank‐sum test (I). Each experiment was repeated independently three times.

To investigate the impact of the gut microbiome on atherosclerosis, 16S rRNA sequencing data were analyzed in fecal samples from chow‐diet‐fed (CD) and HFD‐fed mice, with or without aspirin treatment (**Figure** **1**A). The alpha diversity indices, such as Shannon and Simpson indices, indicated a significant reduction in gut microbiome diversity in HFD groups, which was partially restored by aspirin treatment (Figure 1B). Meanwhile, β‐diversity analysis also showed that the gut microbial composition was substantially reshaped by HFD and aspirin treatment (Figure 1C). Linear discriminant analysis Effect Size (LEfSe) analysis suggested an enrichment of *Ligilactobacillus* and *Lactobacillus* in the treatment of the aspirin group, whose patterns resemble the taxonomy features of the CD group (Figure 1D; Figure S1A, Supporting Information). More interestingly, the microbial composition analysis showed that HFD resulted in shrinkage of *Firmicutes*, *Lactobacillales*, *Lactobacillaceae*, and *Ligilactobacillus* at the levels of Phylum, Order, Family, and Genus, respectively (Figure 1E; Figure S1B–D, Supporting Information), which phenomenon was reversed by aspirin. At the species level, we further found that the abundance of *L. murinus* and *L. johnsonii* was decreased by HFD and restored by aspirin (Figure 1F). On the other hand, the co‐occurrence network analysis revealed weak connections among the core genera in the HFD group compared to the CD group, which were further strengthened in the aspirin group by *L. murinus* and *L. johnsonii* (Figure 1G), indicating the vital roles of both species in resisting HFD.

We also tested this hypothesis in our reported human cohort (Cohort 1^[^ ^6^ ^]^), which included healthy volunteers who took daily oral aspirin for 30 days (Figure 1H). An increase in *Lactobacillus* abundance was observed in this group following aspirin treatment (Figure 1I). Another cohort dataset^[^ ^10b^ ^]^ was used to verify these findings, furthermore (Cohort 2, Figure 1J). This cohort enrolled 275 HC volunteers and 372 patients with IHD, who were categorized into three groups based on diagnosis and drug history of aspirin. Similar to previous findings, the gut composition of *Lactobacillus* was decreased in patients with IHD compared to the HC group, while aspirin users exhibited a higher *Lactobacillus abundance* (Figure 1K). Together, these data indicate that the increase in *L. murinus* and *L. johnsonii* is positively associated with aspirin usage.

### 2.2 *L. Murinus* and *L. Johnsonii* Mediate the Anti‐Atherosclerotic Effect of Aspirin

**Figure 2:**
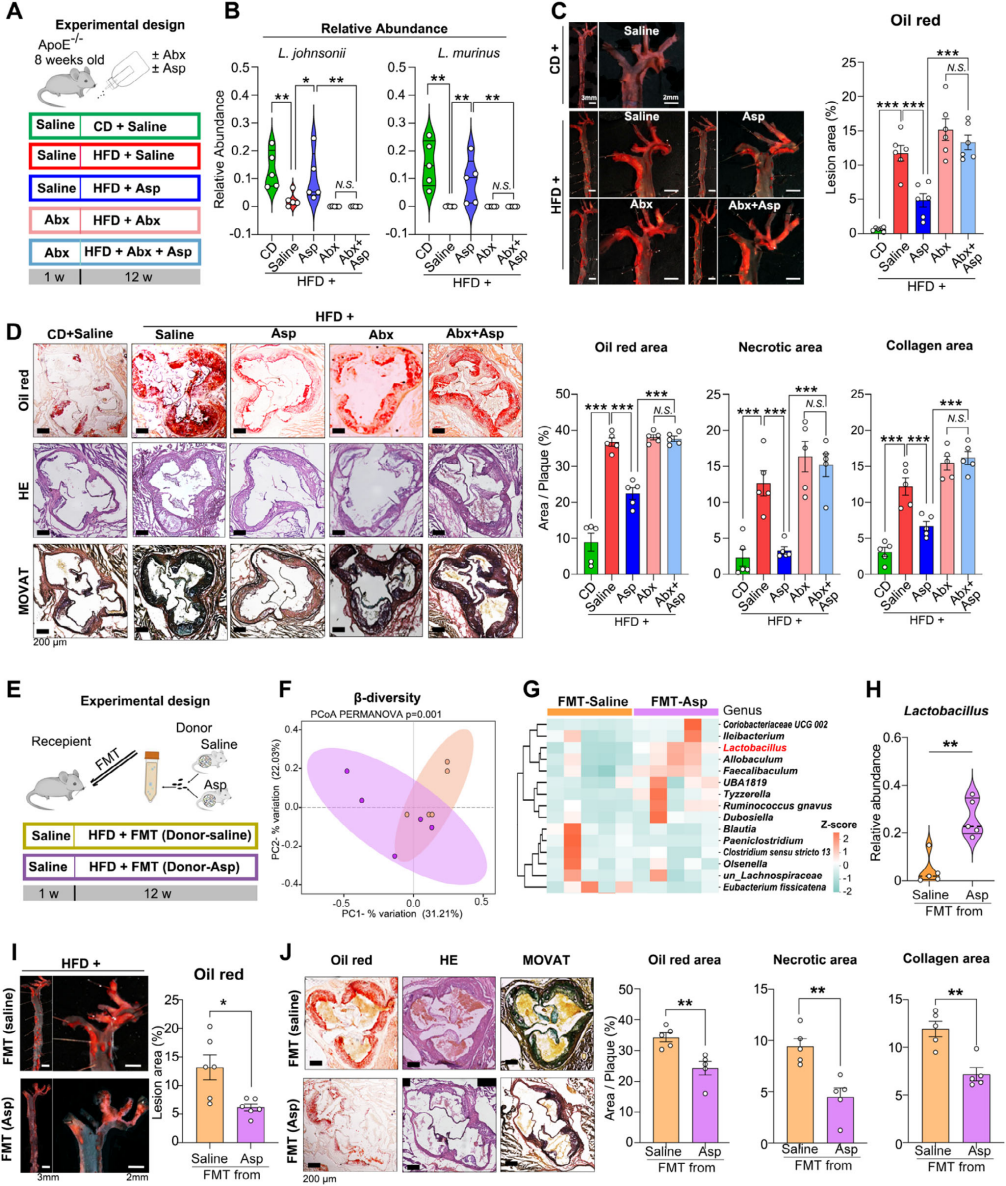
*L. murinus* and *L. johnsonii* mediate the anti‐atherosclerotic effect of aspirin. A) Experimental design: HFD induced aortic atherosclerosis in *ApoE* ^−/−^ mice, which were then treated with aspirin (0.2 mg mL^−1^ in drinking water) and antibiotics (upper set) or vehicle. CD, Chow diet. B) The relative abundance of *L. murinus* and *L. johnsonii* in fecal samples was determined by 16S rRNA sequencing. n = 5/group. C) Representative images of aortas stained with Oil Red O (left) and quantification of the lesions (right). n = 6/group. Scale bar: 3 mm (left), 2 mm (right). D) Left: Representative sections of left ventricular outflow tracts stained with Oil Red O, HE), and MOVAT. Right: The quantification of Oil Red O positive area, necrotic area, and collagen area. Scale bar = 200 µm, n = 5/group. E) Representative images (left) of immunofluorescence staining for CD68 (red), NLRP3 (green), and IL‐1β (pink) in the left ventricular outflow tracts, and the positive areas were statistically analyzed (right). Scale bar: Upper, 200 µm; bottom: 100 µm. n = 5/group. F) The FMT donor mice were treated with HFD together with aspirin (0.2 mg mL^−1^) or saline for 4 weeks before fecal microbiota collection. G) Principal co‐ordinates analysis (PCoA) based on the β‐diversity indexes (bray‐curtis distances) of the two groups. Permutational multivariate analysis of variance (PERMANOVA) test was used. n = 5/group. H) Heatmap of individual genus abundance after FMT from control and asp‐treated mice. n = 5/group. I) Relative abundance of Lactobacillus after FMT from control and asp‐treated mice. J) Left: Representative sections of left ventricular outflow tracts stained with Oil Red O, HE, and MOVAT. Right: The quantification of Oil Red O positive area, necrotic area, and collagen area after FMT from control and asp‐treated mice. Scale bar = 200 µm, n = 5/group. Data are expressed as mean ± s.d. Two tailed Student's t‐test followed by t‐test H, I, J), One‐way ANOVA followed by Fisher's LSD post hoc test (B, C, D). Each experiment was repeated independently three times.

To investigate whether *L. murinus* and *L. johnsonii* mediate HFD‐induced atherosclerosis and their relationship with aspirin, *ApoE* ^−/−^ mice were treated with a CD and HFD, together with aspirin and/or antibiotics (**Figure** **2**A). As expected, the abundance of *L. murinus* and *L. johnsonii* increased after aspirin treatment, but this effect was eliminated by antibiotics (abx) (Figure 2B). Oil Red O staining of the aortas showed that the eradication of gut microbiota by antibiotics attenuated the protective effects of aspirin (Figure 2C,D). Haematoxylin‐Eosin (HE) and Russel‐Movat method (MOVAT) staining also indicated that the necrotic core area and degree of fibrosis were decreased by aspirin, whose effects were rescued by antibiotics (Figure 2D). Body weight and serum lipid concentrations were also examined but were not significantly different among these groups (Figure S1E,F, Supporting Information). It is widely accepted that gut microbial TMAO drives atherosclerosis.^[^ ^8^ ^]^ Therefore, we examined the levels of TMAO and its related metabolites in the serum of our model. However, although antibiotics led to decreased levels of TMAO, no significant changes in TMAO or trimethylamine (TMA) were observed in the aspirin‐treated groups (Figure S1G, Supporting Information).

Moreover, fecal microbiome transplantation (FMT) from aspirin‐ or vehicle‐treated mice to HFD‐fed mice was also conducted (Figure 2E). This was also supported by the results in the FMT model, showing fecal microbiota derived from aspirin‐treated mice helps re‐establish α‐diversity and β‐diversity of the gut microbiota (Figure S2A, Supporting Information; Figure 2F). FMT from aspirin‐treated mice also tended to increase the abundance of *Lactobacillus* (Figure 2G,H), as well as other probiotics, including *Allobaculum*, *Ruminococcus gnavus*, and *Faecalibaculum* (Figure S2B, Supporting Information). More interestingly, FMT from aspirin‐treated mice also reduced plaque burden and necrotic core area in the recipient mice (Figure 2I,J), further suggesting a role of gut microbiota in mediating atherosclerosis.

We have previously reported that pyroptotic macrophages were driving atherosclerosis;^[^ ^16a^ ^]^ thus, we further examined the macrophage marker CD68 and pyroptotic markers, including NLRP3, IL‐1β, in the sections of left ventricular outflows in this model. The results indicated that both aspirin and FMT from aspirin‐treated mice inhibited macrophage infiltration, as well as pyroptosis in HFD‐induced atherosclerotic plaques (Figure S2C, Supporting Information). These effects were found to be diminished by removing gut microbiota through antibiotics. Moreover, the inflammatory markers of aortas, including IL‐6, TNF‐α, and MCP‐1, were also examined by qRT‐PCR, which showed decreased levels in the aspirin group (Figure S2D, Supporting Information). Together, these results suggest that HFD‐induced gut dysbiosis was attenuated by aspirin, potentially through *L. murinus* and *L. johnsonii*.

### 2.3 *L. Murinus* and *L. Johnsonii* Ameliorate HFD‐Induced Atherosclerosis

**Figure 3:**
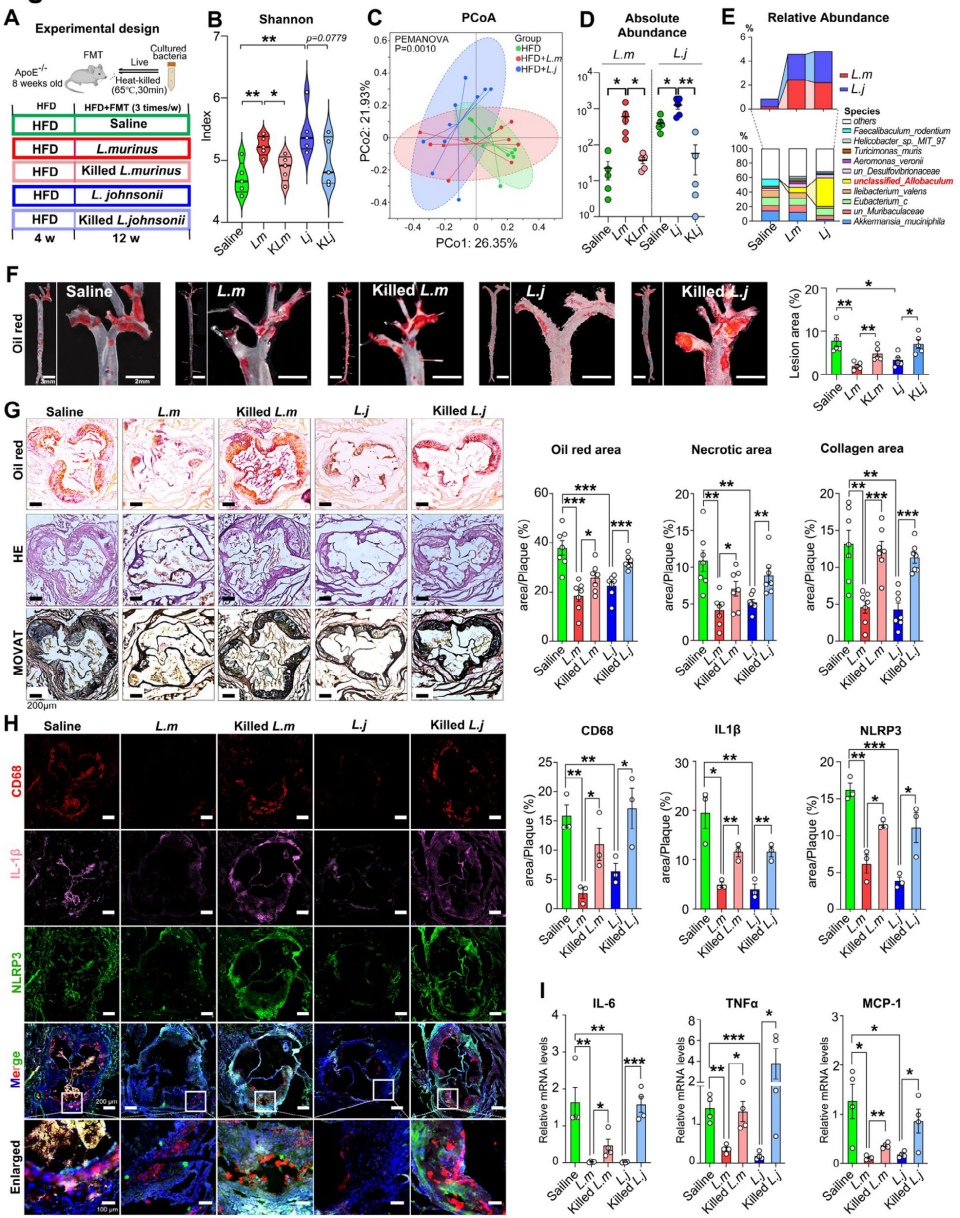
*L. murinus* and *L. johnsonii* ameliorate HFD‐induced atherosclerosis. A) Experimental design showing the transplantation of *L. murinus (L. m)* and *L. johnsonii* (*L. j*) to HFD‐treated *ApoE* ^−/‐^ mice. Supplementation of bacteria (1 × 10^8^ CFU/0.2 mL, 3 times/week) was continued for 12 weeks. Killed *L. m* and *L. j* were prepared by water bath at 65 °C for 30 min before gavage. B) α‐diversity (Shannon index) of the five groups after transplantation of *L. johnsonii* and *L. murinus*. n = 5/group. C) PCoA analysis based on the relative abundances of species of gut microbiota in mice transplantation of *L. johnsonii* and *L. murinus*. PERMANOVA test was conducted in all three groups. D) Absolute abundance of *L. johnsonii* and *L. murinus* after transplantation in the five groups. n = 5/group. E) Top: Relative abundance of *L. johnsonii* and *L. murinus* among the three groups treated as in Figure 3A. Bottom: Relative abundance at species levels of gut microbiota treated as in Figure 3A. F) Representative images of aortas stained with Oil Red O (left) and quantification of the lesion area (Right). n = 5/group. Scale bar:3 mm(left), 2 mm (right). G) Left: Representative sections of left ventricular outflow tracts stained with Oil Red O, HE, and MOVAT. Right: The quantification of Oil Red O positive area, necrotic area, and collagen area. Scale bar = 200 µm, n = 7/group. H) Left: Representative images of immunofluorescence staining for CD68 (red), NLRP3 (green), and IL‐1β (pink) in the aortic roots of mice in each group; Right: the quantification of the positive areas was statistically analysed. Scale bar: upper: 200 µm; bottom: 100 µm. n = 3/group. I) The relative mRNA expression levels of the inflammatory factors, including TNF‐α, MCP‐1, and IL‐6, in the aortas in each group. GAPDH was used as inner control. n = 4/group. Data are expressed as mean ± s.d. One‐way ANOVA followed by Fisher's LSD post hoc test (B, D, F, G, H, I). Each experiment was repeated independently three times.

To further investigate whether the colonization of *L. murinus* and *L. johnsonii* has impacts on HFD‐induced atherosclerosis, both live and heat‐killed strains of these species were utilized in an HFD‐induced model (**Figure** **3**A). The shift of α‐ and β‐diversity induced by HFD was found to be reversed by transplantation of these two live species (Figure 3B,C; Figure S3A, Supporting Information). Interestingly, the increased abundances of *Lactobacillus* and other bacteria, such as *Allobaculum*, *Clostridium*, and *Ruminococcus*, were also found to be identical features in terms of LEfSe analysis and LDA scores (Figure S3B,C, Supporting Information). Meanwhile, the 16S rRNA sequencing data confirmed the successful colonization of both strains (Figure 3D). Consistent with previous findings in aspirin‐modulated microbiome, increased *Allobaculum* was also identified as a characteristic of microbial signature after *L. murinus* and *L. johnsonii* colonization (Figure 3E). Here we showed the top 10 most abundant species (Figure 3E bottom set), as well as the relative abundance of *L. murinus* and *L. johnsonii* (Figure 3E top set). In accordance with the results in Figure 3D, the relative abundance of both strains dramatically increased after colonization. Co‐occurrence network suggested important roles of *L. murinus* and *L. johnsonii* with other core species (Figure S3D, Supporting Information). The correlation results further confirmed a positive correlation between these two genera and other reported probiotics, such as *Allobaculum*, *Escherichia coli* (*E. coli*), *Bacillus subtilis*, as well as *Parabacteroides goldsteinii* (Figure S3E, Supporting Information), and *L. murinus* and *L. johnsonii* have a positive correlation (R = 0.6612, *P*<0.0001) (Figure S3F, Supporting Information). Interestingly, gavage of live *L. murinus* and *L. johnsonii* significantly reduced plaque burden (Figure 3F), necrotic core size, and the degree of fibrosis in HFD‐fed mice (Figure 3G), while heat‐killed strains did not exhibit therapeutic effects. Moreover, live *L. murinus* and *L. johnsonii*, instead of heat‐killed species, inhibited macrophage infiltration and pyroptosis in aortic plaques (Figure 3H). We also observed decreased levels of inflammatory markers such as TNF‐α, IL‐6, and MCP‐1 in the aortas of mice colonized with *L. murinus* and *L. johnsonii* (Figure 3I). Together, these results suggest that *L. johnsonii* and *L. murinus* shift microbiome features and suppress HFD‐induced atherosclerosis.

### 2.4 *L. Murinus* and *L. Johnsonii* are Positively Associated with Butyric Acid

**Figure 4:**
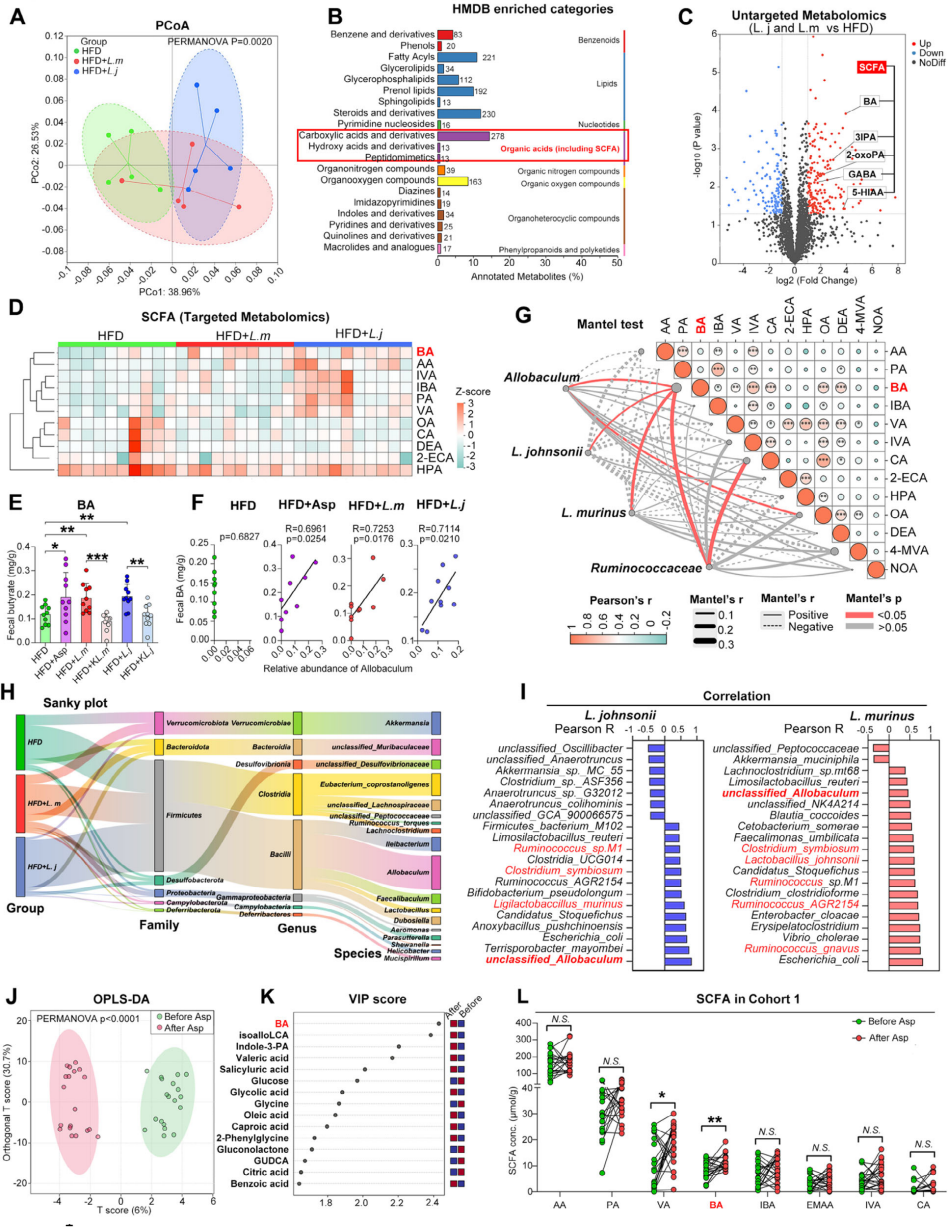
*L. murinus* and *L. johnsonii* promote gut butyric acid production. A) PCoA analysis based on the untargeted metabolomics data derived from HFD‐treated mice after *L. murinus* or *L. johnsonii* transplantation. PERMANOVA test was conducted among the three groups. B) Enriched metabolite pathways in the human metabolome database (HMDB) using the top 20 classes of metabolites after *L. johnsonii* or *L. murinus* transplantation. C) Volcano plot of different metabolites in fecal samples of HFD‐fed mice overlapped after *L. johnsonii* and *L. murinus* transplantation. Red: Metabolites with increased abundance in transplantation group compared with HFD group. Blue: Metabolites with decreased abundance. Grey: Not significant. BA: butyric acid. SCFA: short‐chain fatty acid. 3IPA: 3‐indole propanoic acid. 2‐oxoPA: 2‐oxopropanoate. GABA: γ‐aminobutyric acid. 5HIAA: 5‐hydroxyindole acetic acid. D) Targeted metabolomics detection using GC‐MS/MS method showing heatmap of SCFAs in mice feces as treated in (A). AA, acetic acid; IVA, isovaleric acid; IBA, isobutyric acid; PA, propionic acid; VA, valeric acid; OA, octanoic acid; CA, caproic acid; DEA, decanoic acid; 2ECA, 2‐ethylcaproic acid; HPA, hydroxylpropinoic acid. E) Concentrations of butyric acid (BA) in fecal samples of different groups as treated in Figure 2A and 3A. F) Correlation between relative abundance of *Allobaculum* in stool samples with butyric acid in each group including HFD, HFD + Asp, HFD + *L. m* and HFD + *L. j*. G) Correlations between the SCFAs and four microbial species. Pairwise Pearson's correlation coefficients among the SCFAs are shown. Circle color matches Pearson's correlation. Each species was related to each SCFAs by Mantel tests. Edge width denotes Mantel's r statistic for the corresponding distance correlations. Edge shape and color correspond to the statistical significance of Mantel's r. H) Sanky plot of relative abundance of gut microbiota at family, genus and species levels among the three group. I) Correlation analysis of relative abundance of *L. murinus* and *L. johnsonii* with other species in three groups. J) OPLS‐DA (Orthogonal partial least‐squares discrimination analysis) of the SCFAs levels before and after administration of aspirin in the Cohort 1, N = 19 individual. K) Variable importance in projection (VIP) scores of OPLS‐DA showed the ability of different metabolites to discriminate between groups. A metabolite with VIP score >1.5 was considered important in the discrimination. L) Fecal SCFAs of individuals in Cohort 1 were examined by GC/MS. N = 19 individuals. AA, acetic acid; PA, propionic acid; VA, valeric acid; BA, butyric acid; IBA, isobutyric acid; IVA, isovaleric acid; CA, caproic acid. Data are expressed as mean ± s.d. One‐way ANOVA followed by Fisher's LSD post hoc test (E), PERMANOVA test (J), Paired two‐tailed Student's t test (L), Pearson's R correlation (F, I). Each experiment was repeated independently three times.

Gut microbe regulation of the host is considered to depend on the production of a wide variety of metabolites.^[^ ^18^ ^]^ We then set out to explore the metabolic mechanisms underlying the protective effects of *L. johnsonii* and *L. murinus* against atherosclerosis. Untargeted metabolomic detection using liquid chromatography‐tandem mass spectrometry (LC‐MS/MS) was conducted on fecal samples (**Figure** **4**A–C). The results suggest that both *L. johnsonii* and *L. murinus* transplantation led to a dramatic shift in gut microbial metabolites, as supported by PCoA analysis (Figure 4A). After matching the upregulated metabolites to the Human Metabolites Database (HMDB), an enrichment of organic acids, including SCFAs, was observed (Figure 4B). Moreover, the volcano plot based on untargeted metabolomics also identified several SCFAs, including butyric acid (BA), which were increased dramatically by *L. johnsonii* (Figure 4C). Thus, we further focused on the SCFA profiles through targeted metabolomics detection utilizing gas chromatography‐tandem mass spectrometry (GC‐MS/MS). Fecal butyric acid (BA), along with other SCFAs, were found to be upregulated by *L. murinus* and *L. johnsonii* gavage (Figure 4D). Interestingly, the concentration of lactate, the precursor for the synthesis of butyric acid, was found to be increased in feces after transplantation of *L.johnsonii* and *L.murinus* (Figure S4B, Supporting Information). Besides, aspirin treatment and live bacteria increased gut BA concentrations, while heat‐killed species failed to induce this effect (Figure 4E). A similar trend in butyric acid levels was observed in the serum of the aforementioned groups of mice (Figure S4C, Supporting Information). Interestingly, BA concentrations seemed to be positively correlated with *Allobaculum* when aspirin, *L. murinus*, or *L. johnsonii* were introduced to the gut community, which correlation that was absent in HFD‐fed mice (Figure 4F). Moreover, Mantel tests between species and multiple sets of SCFAs suggest that the intestinal concentrations of BA and related SCFAs were positively correlated with *Lactobacillus*, together with SCFA‐producing bacteria such as *Allobaculum* and *Ruminococcaceae* (Figure 4G). These alterations at the family, genus, and species levels were further verified in Sanky plot assays (Figure 4H). Moreover, *L johnsonii* and *L. murinus* were found to be positively correlated with *Allobaculum* and *Ruminococcus* (Figure 4I).

We further verified these findings in a human cohort. Similar to what was found in the animal model, orthogonal partial least squares discrimination analysis (OPLS‐DA) analysis showed that human gut metabolite spectra were shifted dramatically after aspirin treatment in Cohort 1 (Figure 4J). Interestingly, among these gut microbial metabolites, BA was identified as the top metabolite by variable importance in projection (VIP) score that resulted in the group separation (Figure 4K). The GC/MS‐based targeted SCFA detection was then conducted, which showed increased BA concentrations in the stool samples of Cohort 1 after aspirin treatment (Figure 4L; Figure S4A, Supporting Information). Similarly, concentrations of BA and related SCFAs were positively correlated with *Lactobacillus* and SCFA‐producing genus (Figure S4D,E, Supporting Information). Meanwhile, increased BA concentrations were also detected in the aspirin‐user group in Cohort 2 (Figure S4F, Supporting Information). In an animal model, we found that aspirin treatment could increase the relative abundance of *Allobaculum*. However, other SCFA‐producing species, such as *Faecalibaculum rodentium*, *Ruminococcus gnavus*, *Eubacterium fissicatena*, and *Clostridium cocleatum*, exhibited no significant differences between AS mice treated with HFD and aspirin (Figure S4G, Supporting Information). Together, these results indicated that the treatment of aspirin, *L. murinus*, and *L. johnsonii* may promote gut butyric acid production.

### 2.5 *L. Murinus* and *L. Johnsonii* Promote the Production of Butyrate and Suppress Atherosclerosis Progression

**Figure 5:**
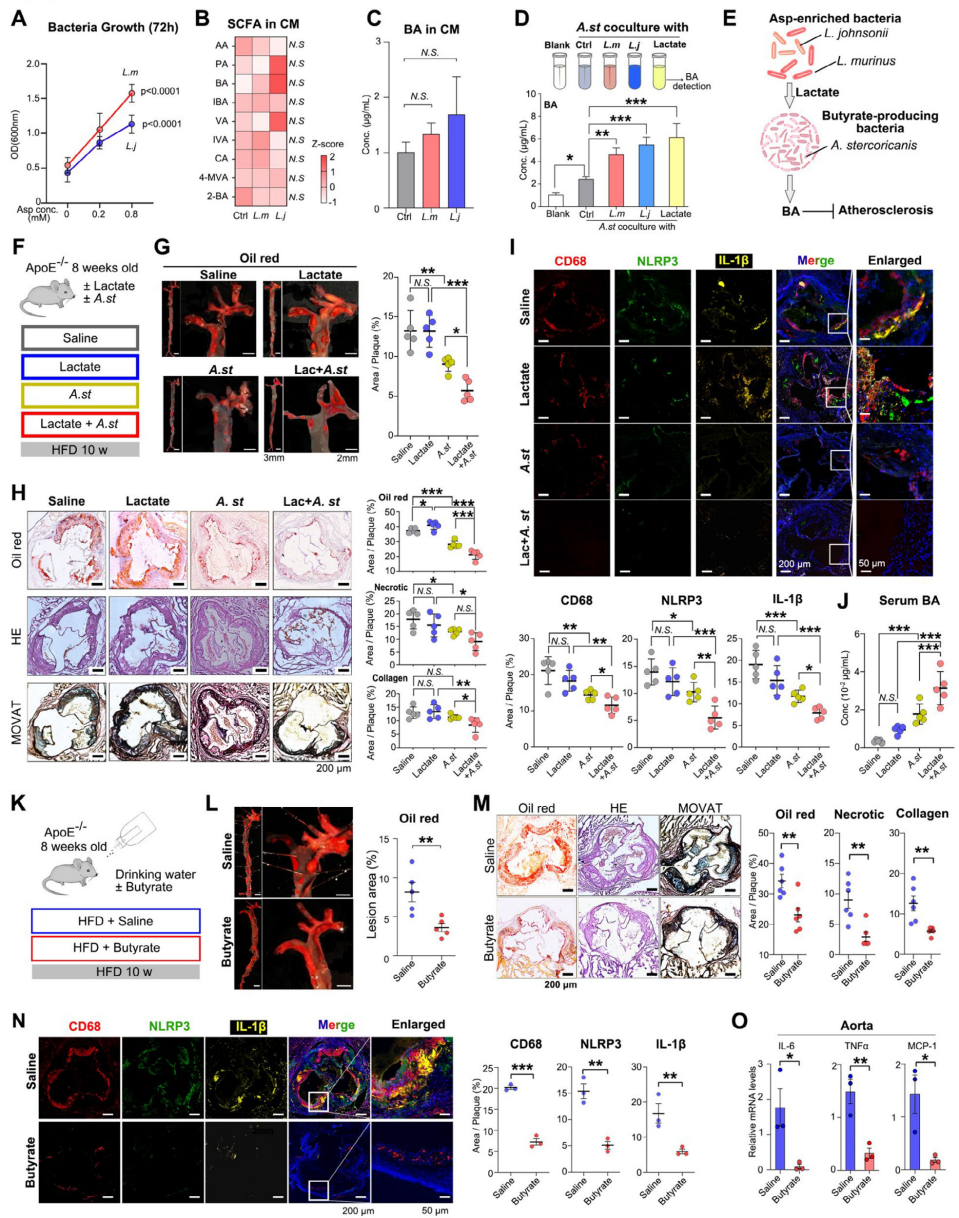
*L. murinus* and *L. johnsonii* facilitate *Allobaculum's* production of butyrate and inhibits atherosclerosis. A) Aspirin treatment promotes bacteria growth of *L. murinus* and *L. johnsonii* for 72 h in vitro. Statistical analysis was conducted between [Asp concentration = 0.8 mM] group with [concentration = 0 mM] group. n = 5/group. B) Upper CM was subjected to GC‐MS to detect the SCFA concentrations. Blank culture medium with no bacteria being cultured were used as control group. AA, acetic acid; PA, propionic acid; BA, butyric acid; IBA, isobutyric acid; VA, valeric acid; IVA, isovaleric acid; CA, caproic acid; 4‐MVA, 4‐methylvaleric acid; 2‐BA, 2‐butyric acid. n = 3/group*. N.S, P>0.05*. C) BA concentrations in upper CM of *L. murinus* and *L. johnsonii*. n = 3/group. D) In vitro cultures of *A. stercoricanis* were co‐cultured with *L. murinus* or *L. johnsonii* (OD = 0.6) for 24 h. Upper conditioned media were then detected for butyrate. E) Butyrate‐producing pathway: Asp‐enriched *L. murinus* and *L. johnsonii* provide lactate in the gut, which promote the expansion of butyrate‐producing bacteria including *Allobaculum* and facilitate the production of butyrate from lactate. F) Experimental design involves *ApoE^−/−^* mice that are treated with or without lactate and colonized with or without *A. stercoricanis*. G) Representative images of aortas stained with Oil Red O (left) and quantification of the lesions area (Right). n = 5/group. Scale bar:3 mm(left), 2 mm (right). H) Left: Representative sections of left ventricular outflow tracts stained with Oil Red O, HE and MOVAT. Right: The quantification of Oil Red O positive area, necrotic area and collagen area. Scale bar = 200 µm, n = 5/group. I) Top: Representative images of immunofluorescence staining for CD68 (red), NLRP3 (green), and IL‐1β (yellow) in the aortic roots of mice in each group; Bottom: the quantification of the positive areas was statistically analysed. Scale bar: upper: 200 µm; bottom: 50 µm. n = 3/group. J) Serum butyrate concentrations were detected in mice treated as with or without lactate, with or without colonization of *A. stercoricanis*. n = 5/group. K) Experimental design of HFD‐fed *ApoE* ^−/−^ mice treated with vehicle or butyrate. L) Representative images of aortas stained with Oil Red O (left) and quantification of the lesions (Right). n = 5/group. Scale bar:3 mm(left), 2 mm(right). M) Left: Representative sections of left ventricular outflow tracts stained with Oil Red O, HE and MOVAT. Right: The quantification of Oil Red O positive area, necrotic area and collagen area. Scale bar = 200 µm, n = 6/group. N) Left: Representative images of immunofluorescence staining for CD68 (red), NLRP3 (green), and IL‐1β (yellow) in the left ventricular outflow tracts of each group. Right: the positive areas were statistically analysed. Scale bar: upper, 200 µm; bottom, 50 µm. n = 3/group. O) The relative mRNA expression levels of the inflammatory factors including TNF‐α, MCP‐1 and IL‐6 in the aortas of each group. n = 3/group. Data are expressed as mean ± s.d. One‐way ANOVA followed by Fisher's LSD post hoc test (C, D, G, H, I, J). Two tailed student t test (A, L, M, N, O). Each experiment was repeated independently three times.

We then explored how *L. murinus* and *L. johnsonii* enriched in aspirin‐treated mice facilitate butyrate production. First, we observed that a 72 h treatment with aspirin promoted the growth of *L. murinus* and *L. johnsonii* in vitro (**Figure** **5**A). However, the upper culture medium (CM) of *L. murinus* and *L. johnsonii* turned out to show no significant increase in BA, compared to the blank CM (Figure 5B,C), which excluded the suspected possibility that both species are BA producers. In vitro assays suggest that co‐culturing with either *L. murinus* or *L. johnsonii* tended to increase the BA production in the CM of *Allobaculum*, further suggesting the beneficial effects of *L. murinus* and *L. johnsonii* in maintaining the BA‐producing microbial community (Figure 5D). Interestingly, we also found that adding lactate to the culture medium of *Allobaculum* also increased BA production (Figure 5D). Recent reports have indicated bacterial synthetization of butyrate using lactate.^[^ ^19^ ^]^ Thus, we hypothesized that *L. murinus* and *L. johnsonii* may provide lactate to butyrate‐producing bacteria such as *Allobaculum*, which facilitate the production of butyrate from lactate (Figure 5E). We then tested this hypothesis in vivo by treating HFD‐fed mice with lactate or without lactate, with or without the transplantation of *A. stercoricanis* (Figure 5F). Interestingly, in HFD‐fed mice treated with lactate, only the colonization of *A. stercoricanis* exhibited therapeutic effects in preventing atherosclerosis compared to the non‐colonized group. This treatment also resulted in a reduction of necrotic core size and the degree of fibrosis in plaques at the left ventricular outflow. Notably, compared to the independent colonization of *A. stercoricanis*, which also contributed to the effect. Lactic acid in conjunction with *A. stercoricanis* further reduced the burden of atherosclerotic plaques (Figure 5G,H). Meanwhile, the percentages of CD68‐, NLRP3‐, and IL‐1β‐positive areas in plaques were detected, which showed that *A. stercoricanis* inhibited macrophage infiltration and pyroptosis (Figure 5I). However, the intervention of lactate alone did not exhibit similar therapeutic effects. One of the possible explanations may be the limited increase in serum butyrate (Figure 5J) without the transplantation of *A. stercoricanis*, an important BA producer.

To further determine the effects of microbial butyrate on atherosclerosis development, HFD‐fed *ApoE* ^−/−^ mice were treated with butyrate, the ionized form of butyrate dissolved in drinking water (Figure 5K). The results showed the therapeutic effects of butyrate in preventing atherosclerosis (Figure 5L,M) and in inhibiting macrophage infiltration and pyroptosis (Figure 5N). The mRNA expression levels of inflammatory factors, such as TNF‐α, MCP‐1, and IL‐6 in the aortas also showed similar changes (Figure 5O). Together, these results indicate a symbiotic relationship between *Lactobacillus* and *Allobaculum* in producing BA, which suppresses inflammatory signals and the progression of atherosclerosis.

### 2.6 Butyrate Attenuates HFD‐Damaged Gut Barrier and LPS‐Induced Macrophage Pyroptosis

**Figure 6:**
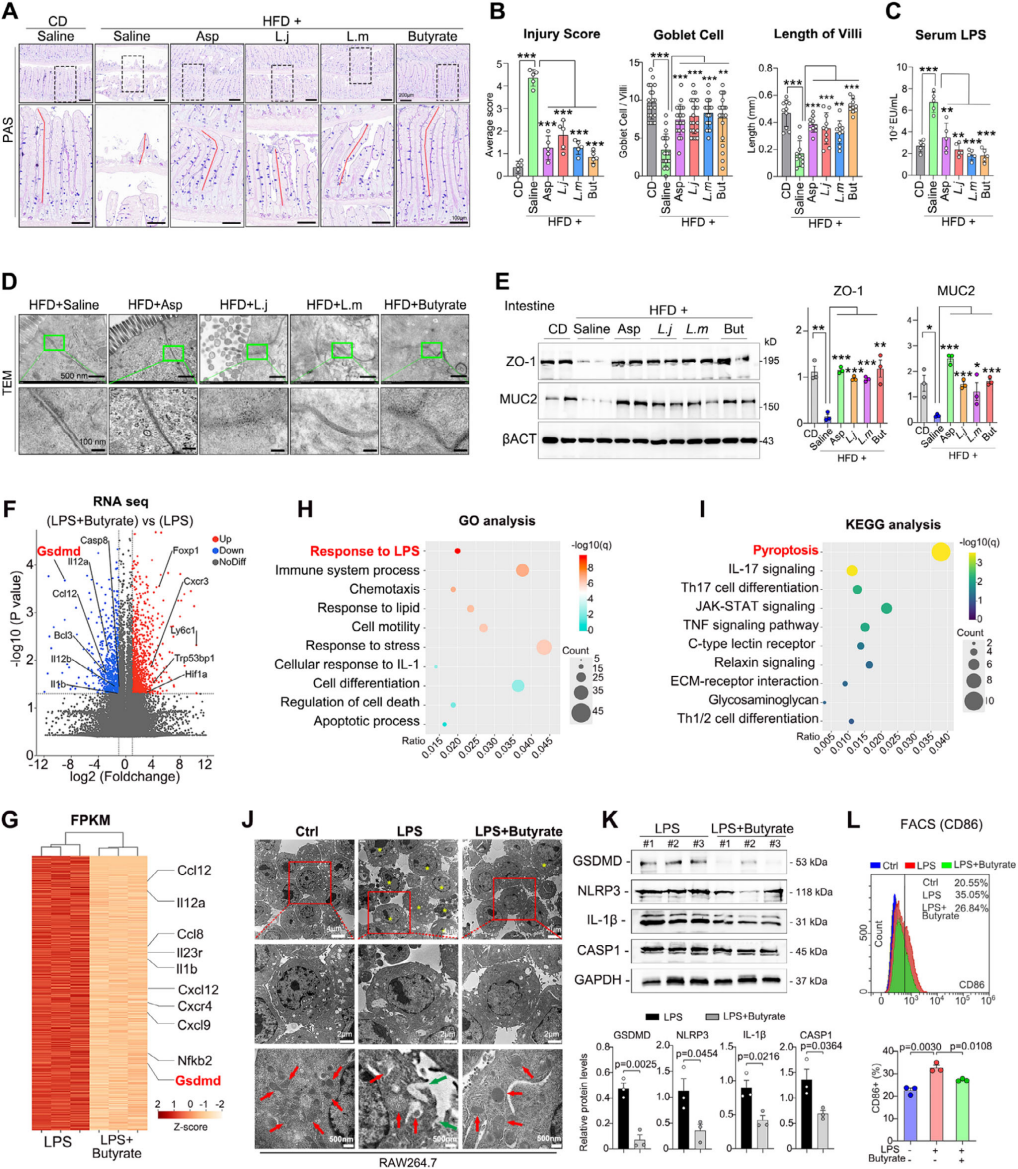
Butyrate attenuates HFD‐damaged gut barrier and LPS‐induced macrophage pyroptosis. Representative images of Periodic Acid‐Schiff (PAS) and Alcian Blue (AB) staining of intestine from mice fed a CD, HFD, and those supplemented with Asp, along with *L. m*, *L. j*, and butyrate, are presented as described in Figures 3A and 5K. Scale bars: 200 µm (upper set) and 100 µm (lower set). n = 5/group. The numbers of goblet cells, and the length of intestinal villus to valuate gut barrier (n = 5/group). Intestinal injury scores of mice were evaluated by Chiu's scoring system. Random 5 positions of distal jejunum were picked and average scores calculated for all the mice. Goblet cell number per villi were statistically analysed. Random 20 visions of distal jejunum were picked and average numbers per villi were calculated for all the mice. Length of Villi were statistically analysed. Random 10 visions of distal jejunum were picked and average numbers per villi were calculated for all the mice. Serum LPS concentration in each group was statistically analysed. (n = 5/group). Representative images using TEM showing the tight junction of intestinal epithelium of HFD‐treated mice as in (A). The protein expression levels of intestinal ZO‐1 and MUC2 in mice treated with aspirin, *L. murinus*, *L. johnsonii* and butyrate, as described in Figures 2A and 3A and 5K. β‐ACTIN was used as inner control. RNA sequencing was conducted in RAW264.7 cells upon LPS treatment with or without butyrate. Volcano plots showed the differentially expressed genes (DEG) between [LPS + butyrate] and [LPS] group. DEGs were identified by a fold change > 2 and adjust *P* value < 0.05. n = 3/group. DEGs were shown by a cluster heatmap according to FPKM analysis. GO analysis based on downregulated genes in [LPS + butyrate] group (compared with LPS group) were analysed and top10 most enriched pathways were showed. KEGG analysis based on downregulated genes in [LPS + butyrate] group (compared with LPS group) were analysed and top10 most enriched pathways were showed. TEM showed RAW264.7 cells after LPS treatment with or without butyrate. Yellow asterisk: pyroptotic macrophages; Red arrow: mitochondria; Green arrow: pores on cell membrane. Butyrate inhibited the expression of GSDMD, NLRP3, IL‐1β and Casepase‐1 in peritoneal macrophage induced by LPS. Flow cytometry showed the CD86‐positive percentage of RAW264.7 cells upon LPS stimulation with or without butyrate treatment.Data are expressed as mean ± s.d. Two tailed student t test (K) and One‐way ANOVA (B, C, E, L) followed by Fisher's LSD post hoc test. Each experiment was repeated independently three times.

HFD‐induced leaky gut has been identified as one of the contributing factors to atherosclerosis. To elucidate the mechanisms underlying the therapeutic effects of *L. murinus* and *L. johnsonii*, we explored the integrity of the gut barrier following the transplantation of both strains. Damaged gut barrier and increased injury scores were observed in HFD‐fed mice, compared with CD‐fed mice (**Figure** **6**A). Interestingly, treatment with aspirin or butyrate, as well as the transplantation of either *L. murinus* or *L. johnsonii*, all preserved the gut barrier, increased the number of goblet cells, and the length of intestinal villus (Figure 6B). More importantly, the serum LPS concentrations increased by HFD were also reversed by treatment with aspirin, both probiotics and butyrate (Figure 6C). Transmission electron microscopy (TEM) also suggested that aspirin, these two stains, as well as butyrate, preserved the tight junction of the intestinal epithelium, which was damaged by HFD (Figure 6D). The potential mechanism may involve increasing the protein levels of the tight‐junction markers ZO‐1 and MUC2 (Figure 6E). These results suggest that *L. murinus* and *L. johnsonii* may inhibit atherosclerosis by preserving the gut barrier and reducing serum LPS levels.

To elucidate the downstream mechanisms underlying butyrate's inhibitory effects on inflammation, RNA sequencing was conducted in peritoneal macrophages upon LPS and butyrate treatment (Figure 6F). Compared to control cells, butyrate was found to suppress many atherosclerosis‐associated inflammatory cytokines,^[^ ^20^ ^]^ including IL‐1β, Ccl12, IL‐12α/β, and Bcl3, etc (Figure 6G). Among the genes with decreased expression, IL‐1β and GSDMD were noticed as key effectors of the pyroptosis signaling pathway. Moreover, GO analysis of downregulated genes was found to be enriched in pathways against LPS and related inflammation (Figure 6H). Similarly, KEGG analysis suggests that butyrate inhibits pyroptosis, TNF‐α, and JAK‐STAT pathways (Figure 6I). TEM also showed that LPS‐induced pyroptosis of macrophages and mitochondrial swelling was suppressed by butyrate (Figure 6J). Furthermore, we verified that butyrate inhibited the expression of NLRP3, Caspase‐1, GSDMD, and IL‐1β in LPS‐treated macrophages (Figure 6K), all of which were proven to drive pyroptosis in macrophages.^[^ ^21^ ^]^ Additionally, flow cytometry assays revealed a significant decrease in the proportion of M1 macrophages (CD86‐positive) following butyrate treatment (Figure 6L). Collectively, these findings suggest that butyrate mitigates atherosclerosis by suppressing LPS‐induced GSDMD‐dependent pyroptosis in macrophages.

### 2.7 Butyrate Inhibits Macrophage Pyroptosis through Activating GPR109A

**Figure 7:**
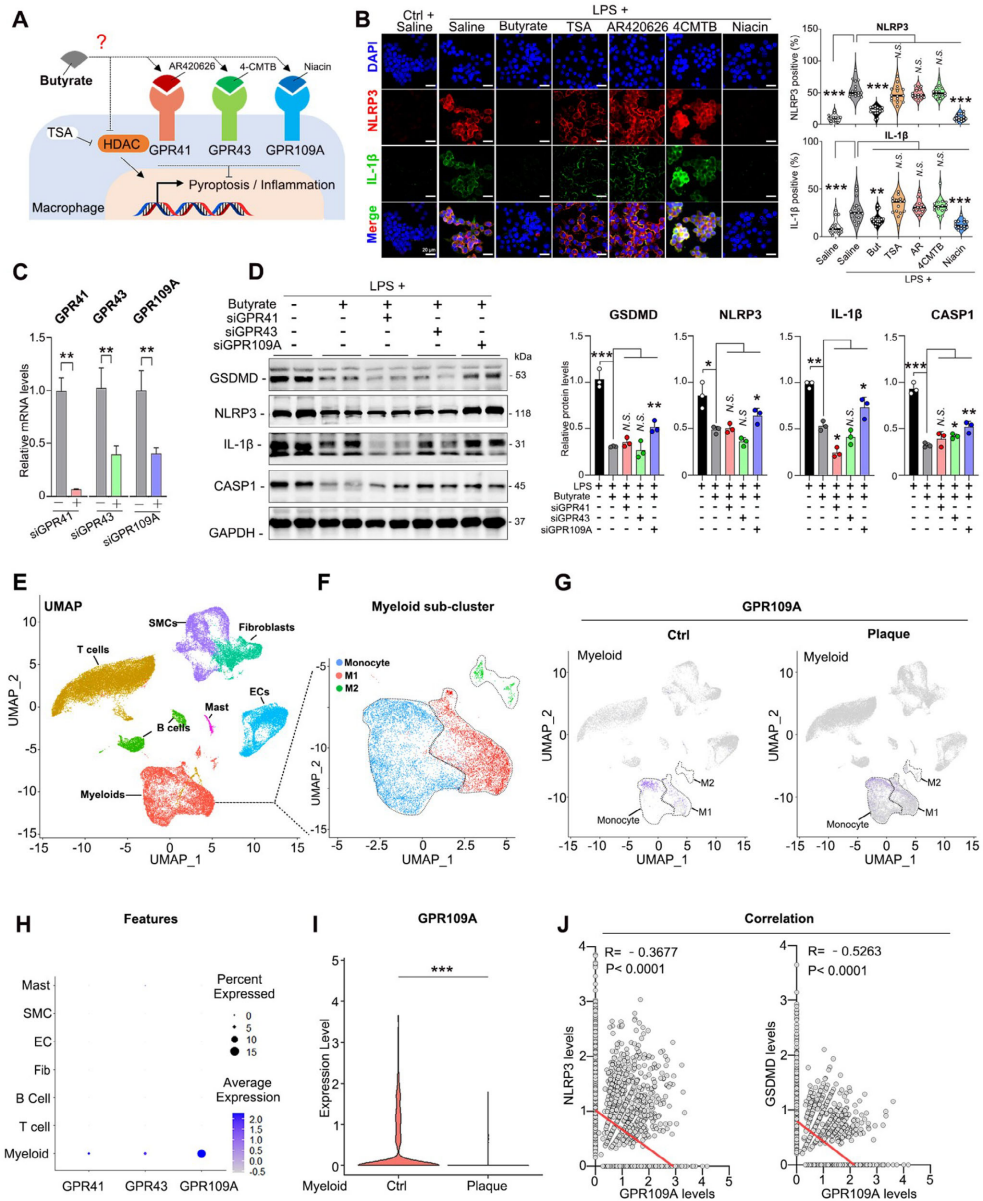
Butyrate inhibits macrophage pyroptosis through activating GPR109A. A) Schematic model of downstream pathways of butyrate and their agonist or antagonist. HDAC, histone deacetylases; TSA, Trichostatin A, a HDAC antagonist; AR420626, GPR41 agonist; 4‐CTMB, (S)‐2‐(4‐chlorophenyl)‐3‐ methyl‐N‐(thiazol‐2‐yl)‐butanamide, GPR43 agonist, Niacin, GPR109A agonist. B) Representative images of immunofluorescence staining for NLRP3 and IL‐1β in macrophage upon LPS stimulation and treatment of butyrate, TSA, AR42026, 4‐CTMB and Niacin. Scale bar: 20 µm, n = 15/group. C) qRT‐PCR results showed the GPR41/43/109A mRNA levels after siRNA transfection in RAW 264.7 cells. GAPDH was used as inner control. D) he expression of GSDMD, NLRP3, IL‐1β and Caspase‐1 in LPS‐treated RAW264.7 cells after siRNA transfection and butyrate treatment. GAPDH was used as inner control. E) Single‐cell sequencing (scSeq) data were obtained from GEO by the dataset (GSE159677) produced by Alsaigh et al. The dataset contains the single‐cell transcriptome of atherosclerotic core plaques (Plaque group) and patient‐matched proximal adjacent portions of carotid artery tissue from patients undergoing carotid endarterectomy (Ctrl group). Uniform manifold approximation and projection (UMAP) distribution of clustering revealed seven distinct cell populations. Population identities were determined based on marker gene expression. SMCs, smooth muscle cells; ECs, endothelial cells. F) UMAP distribution of clustering revealed distinct monocyte, M1 and M2 cell populations. Population identities were established based on the expression of specific marker genes. G) A dot plot showing GPR41, GPR43 and GPR109A expression levels in different types of cells. H) Biaxial scatter plots showing the expression pattern of GPR109A among different subgroups of aorta cells. I) The differential expression of GPR109A between control and plaque group by biaxial scatter plot and statistical analysis. J) Correlation analysis between GPR109A, NLRP3 and GSDMD. Data are expressed as mean ± s.d. Two tailed student t test (C, I) and One‐way ANOVA (B, D) followed by Fisher's LSD post hoc test. Pearson's R correlation (J). Each experiment was repeated independently three times.

It is widely accepted that SCFAs regulate cellular biology mainly by activating their natural receptors, including G protein‐coupled receptors 41, 43, and 109A (GPR41, GPR43, and GPR109A),^[^ ^22^ ^]^ or by inhibiting the activity of histone deacetylase (HDAC).^[^ ^23^ ^]^ We then set out to explore the downstream signaling pathways of butyrate in macrophages (**Figure** **7**A). Notably, both butyrate and agonists of GPR109A inhibited the expression of NLRP3 and IL‐1β in LPS‐treated macrophages, while the HDAC inhibitor and GPR41/43 agonist did not induce similar changes (Figure 7B). To further confirm which receptor was responsible for the downstream effects mediated by butyrate, siRNAs targeting these receptors were subsequently applied (Figure 7C). Interestingly, knockdown of GPR109A, as opposed to other receptors, partially abrogated the butyrate‐induced inhibition of GSDMD, NLRP3, and IL‐1β (Figure 7D). These results indicate that GPR109A in macrophages mediates the anti‐pyroptosis effects of butyrate. To assess the clinical relevance of this regulation, single‐cell sequencing data from aortas containing atherosclerotic plaques were obtained from the Gene Expression Omnibus (GEO) under the dataset (GSE159677) produced by Alsaigh et al.^[^ ^24^ ^]^Clustering analysis utilizing Uniform Manifold Approximation and Projection (UMAP) revealed eight major clusters of aortic cells within this dataset (Figure 7E; Figure S5A, Supporting Information). Clusters of monocytes, as well as M1 and M2 cell populations, are illustrated in Figure 7F. The characteristics of GPR41, GPR43, and GPR109A were examined, revealing that the expression of GPR109A was relatively higher in myeloid cells compared to the other two receptors (Figure 7G). Interestingly, the expression of GPR109A was found to be decreased in atherosclerotic macrophages compared to the control group (Figure 7H,I). In contrast, a slight increase in the expression of GPR41 was identified in plaque (Figure S5B,C, Supporting Information), while no significant change was observed in the expression of GPR43 (Figure S5D,E, Supporting Information). More importantly, the expression of GPR109A was found to be negatively correlated with the expression of pyroptosis markers, including NLRP3 and GSDMD (Figure 7J). However, no correlation was found between the expression of IL‐1β and GPR109A in myeloid cells (Figure S5F, Supporting Information). Further analysis revealed that the expression level of GPR109A in monocytes was higher than in M1 and M2 cells. Additionally, when comparing the control group to the plaque group, it was observed that the expression of GPR109A in monocytes and M1 cells decreased, while no change was detected in M2 cells (Figure S5G,H, Supporting Information). Furthermore, in atherosclerotic plaques, the expression of GPR109A in monocytes and M1 cells, which may play a pro‐inflammatory role, was lower than that in the normal group. In contrast, M2 cells, which are thought to exert anti‐inflammatory effects, showed no significant change in GPR109A expression between the plaque group and the control group. This phenomenon suggests that GPR109A may play an anti‐inflammatory role in regulating macrophage activation and polarization, thereby inhibiting the progression of atherosclerosis. Together, these data demonstrate that GPR109A may play a key role in mediating the inhibitory effects of butyrate on the pyroptosis of intraplaque macrophages.

## 3 Discussion

In this study, we showed a gut microbiota‐butyric acid axis modulating macrophage pyroptosis and atherosclerosis (Figure S6, Supporting Information). This axis involves increased abundance of probiotics and facilitated gut microbial production of butyric acid from lactate, which inhibits macrophage pyroptosis, preserves the gut barrier, and suppresses the progression of atherosclerosis. It has been one of the most exciting findings that many commonly used non‐antibiotic drugs—such as statins and metformin—change microbiome patterns and function.^[^ ^25^ ^]^ The interaction between gut microbes and drugs is bidirectional: gut microbiome composition can be influenced by drugs, but, vice versa, the gut microbiome can also influence an individual's response to a drug by enzymatically transforming the drug's structure.^[^ ^26^ ^]^ As one of the most commonly used oral drugs, aspirin is drawing a lot of attention considering its potential interaction with the gut microbiome. One recent study showed that gut microbiota degrades aspirin and reduces its chemopreventive effects against colorectal cancer.^[^ ^27^ ^]^ But few studies have focused on the effects of aspirin on gut microbiota. Although several studies indicate some correlation between the use of aspirin and change in microbial composition,^[^ ^25d^ ^]^ the direct regulation of aspirin on specific species of microbiota remains obscure. Recently, we reported that the use of aspirin results in a shift of gut microbial spectrum in an animal model of aspirin‐induced intestinal injury.^[^ ^6^ ^]^ We found that the abundance of *Parabacteroides* was significantly reduced by aspirin, which led to insufficiency of bile acids and damaged intestinal stem cell function. In our group's previous research,^[^ ^6^ ^]^ ordinary mice were administered high‐dose aspirin for a brief duration of two weeks to establish an aspirin‐induced intestinal injury model. And in our atherosclerosis model, prolonged feeding of *ApoE^−/‐^* mice with a high‐fat diet can lead to damage in the intestinal tract. Additionally, long‐term low‐dose aspirin may modify the composition of the intestinal microbiota, which could facilitate the repair of the intestinal barrier. However, it remains unexplored whether and how the changes of microbial composition function in the context of atherosclerosis progression.

Various studies have proven that the progression of ASCVD is driven by gut dysbiosis, including increased abundance of *Collinsella*, *Enterobacteriaceae*, and decreased abundance of *Ruminococcaceae* and *Eubacterium*.^[^ ^28^ ^]^ In this study, we also found that HFD induced gut dysbiosis in *ApoE* ^−/−^ mice, which is consistent with previous findings.^[^ ^29^ ^]^ Interestingly, we further found that treatment with aspirin helped the gut microbial community recover from the dysbiosis led by HFD. This process may be through increasing the abundance of two species, *L. murinus* and *L. johnsonii*, which belong to the same Order of *Lactobacillales* and are Gram‐positive, facultative anaerobic bacteria. This result is in line with previous findings of us and the other group, showing that aspirin increases the abundance of genus *Lactobacillus*.^[^ ^6^, ^27^ ^]^

*L. johnsonii* primarily colonizes the human intestinal tract and may exhibit bile salt hydrolytic (BSH) activity^[^ ^30^ ^]^ as well as anti‐inflammatory and immunomodulatory effects.^[^ ^31^ ^]^ It can also activate calcium channels,^[^ ^32^ ^]^ thereby regulating calcium metabolism. In contrast, *L. murinus* primarily colonizes the intestines of rodents and is relatively scarce in the human intestine. Previous studies have demonstrated that *L. murinus* can activate the PPAR‐γ signal pathway^[^ ^33^ ^]^ and modulate GABA levels,^[^ ^34^ ^]^ thereby influencing intestinal lipid metabolism. In our study, both *L. murinus* and *L. johnsonii* were found to enhance butyric acid production and exhibit anti‐atherosclerotic effects. Additionally, we observed a positive correlation between these two genera and other reported probiotics, such as *Allobaculum*, *Escherichia coli* (*E. coli*), and *Bacillus subtilis* (Figure S3E,F, Supporting Information).

In our study, both *L. murinus* and *L. johnsonii* strains exhibited a significant increase following colonization. However, the changes in relative abundance observed were less pronounced than those illustrated in Figure 3D. One possible explanation for this discrepancy is that the overall expansion of the gut microbiota may have diluted the dominant presence of these two bacterial species after colonization.

Our further results suggest that the eradication of gut microbiota by antibiotics abrogated aspirin's protective effects, while FMT from aspirin‐treated mice successfully attenuated the HFD‐induced atherosclerosis. One recent multi‐omics analysis of gut microbiome and clinical outcome also suggests that the influences of aspirin on gut flora have a great impact on the progression of disease, such as diabetes, coronary artery disease, and heart failure.^[^ ^25d^ ^]^ Our results provided more mechanical explanations on how these changes of microbiota mediate the anti‐atherosclerotic function in the context of aspirin's pharmacological actions.

Atherosclerosis is a chronic disease closely related to inflammation and metabolism,^[^ ^12^ ^]^ which is modulated by gut microbiota through their metabolites.^[^ ^10a^ ^]^ Here we also found that the anti‐atherogenic effects of aspirin and these two probiotics had little effect on serum lipids and TMAO, the best‐known gut flora metabolite that deteriorates atherosclerosis.^[^ ^8^ ^]^ Thus, other metabolites produced by these aspirin‐related microbiota were then explored for their potential function in this model. The gut microbial metabolites SCFAs are currently regarded as key regulators in intestinal homeostasis, organ metabolism, inflammation, and immunity.^[^ ^22^, ^35^ ^]^ Interestingly, our metabolomic analysis suggests that the signature of SCFAs was changed by aspirin treatment and gavage of *L. murinus* or *L. johnsonii*. These results are consistent with our previous findings, indicating the expansion of the SCFAs pool after aspirin treatment.^[^ ^6^ ^]^ The cohort‐based results in this study also support the relationship between the use of aspirin and the change in intestinal SCFAs. However, neither *L. murinus* nor *L. johnsonii* can produce SCFAs a lack of necessary enzymes for synthesis. This was also verified in our in vitro assays quantifying the concentrations of SCFAs in their culture medium them. Thus, the correlation between *Lactobacillus* and other SCFA‐producing bacteria may possibly explain the reasons why these interventions help recover the SCFA pool. It is noted that the proportion of several SCFA‐producing species, such as *Allobaculum* and *Ruminococcus*,^[^ ^22^, ^36^ ^]^ was found to expand after *L. murinus* or *L. johnsonii* colonized in this study. *Allobaculum* is generally low in abundance in the gut microbiota of healthy humans and is not considered a dominant genus.^[^ ^37^ ^]^ Several studies have observed changes in *Allobaculum* abundance in patients with obesity and type 2 diabetes.^[^ ^38^ ^]^ By contrast, *Allobaculum* is a prevalent genus in the gut microbiota of mice, particularly in standard laboratory strains (e.g., C57BL/6).^[^ ^39^ ^]^ It may contribute to the production of SCFAs and play a role in host metabolic regulation and anti‐inflammatory effects. In our study, it could exert anti‐atherosclerosis and inhibit macrophage pyroptosis. Further, the in vitro model demonstrated that co‐culturing *L. murinus* or *L. johnsonii* with *Allobaculum* resulted in increased production of SCFAs. One possible explanation for this phenomenon is that the lactate provided by *L. murinus* or *L. johnsonii* serves as a crucial precursor for SCFAs, including butyric acid.

Lactate, the final product of glycolysis, has been widely considered a trigger of inflammation.^[^ ^40^ ^]^ A cross‐sectional study involving 1496 participants reported a positive correlation between serum lactate levels and carotid atherosclerosis.^[^ ^41^ ^]^ Research has also demonstrated that lactic acid can promote vascular inflammatory responses associated with atherosclerosis.^[^ ^42^ ^]^ Thus, the negative effects of lactate on HFD‐induced atherosclerosis may be attributed to promoting endothelial injury and inflammation. In our in vivo study, the introduction of *Allobaculum* into the gut community resulted in the rapid conversion of lactate into butyrate. Notably, the combination of *A. stercoricanis* and lactate produced the most significant reduction in plaque and suppression of pyroptosis. Correspondingly, this combination resulted in the critical role of *Allobaculum*‐mediated conversion of pro‐inflammatory lactate to anti‐inflammatory butyrate in mitigating HFD‐induced atherosclerosis.

SCFAs derived from gut flora have been previously found to ameliorate the HFD‐induced atherosclerosis in an animal model.^[^ ^10a^ ^]^ But the underlying mechanism hasn't been explained. Here, we explored the multiple beneficial functions of butyrate on host physiology in terms of the gut‐artery axis. First, in vivo and in vitro results showed that butyrate can exert anti‐inflammatory and anti‐atherogenic effects by reducing macrophage pyroptosis. As one of the important types of programmed cell death, macrophage pyroptosis has been considered as one of the hallmarks of atherosclerosis.^[^ ^14^ ^]^ Pyroptotic cell death is characterized by assembly of the NLRP3 inflammasome leads to caspase 1‐dependent release of the pro‐inflammatory cytokines IL‐1β and IL‐18 and GSDMD‐mediated pore formation.^[^ ^14^ ^]^ In this study, we found that treatment with aspirin, probiotics, and butyrate all decreased the expression of intraplaque pyroptosis markers, including NLRP3 and IL‐1β, as well as infiltration of macrophages. These results suggest promising probiotics strategies in the prevention of macrophage pyroptosis and ASCVD. We further identified GPR109A as the downstream receptor of butyric acid by in vitro macrophage‐based assays. This was also verified by our reanalysis of single‐cell sequencing data in human aortas, which showed the inverse correlation between butyrate receptor GPR109A levels and pyroptosis markers. More interestingly, we also found that expression of GPR109A was more prevalent in the M0 subset, while macrophages in the M1 sub‐cluster showed decreased expression of it (Figure S5G,H, Supporting Information). Previous studies^[^ ^7^ ^]^ have confirmed that the activation of GPR109A in monocytes can inhibit pyroptosis. Our research further indicates that GPR109A exerts anti‐inflammatory effects by regulating the activation and polarization of macrophages. Further experiments are necessary to investigate the downstream signaling pathway that links GPR109A to GSDMD‐dependent pyroptosis in macrophages. However, the other two receptors for SCFAs, GPR41 and GPR43, also exhibit some anti‐inflammatory effects in vitro, which should be explored in future studies. The other beneficial influence of butyric acid in preventing atherosclerosis——its protection on gut barrier——is also observed in this study. It has been reported that the intestinal barrier protection of butyrate is involved in the process of preventing leakage of enterotoxin and inflammatory factors.^[^ ^43^ ^]^ Consistent with these findings, our study showed that butyrate treatment alleviated the damaged gut barrier resulting from HFD. Similar results were also observed with low‐dose aspirin treatment and the administration of probiotics via gavage, further providing evidence that aspirin protects arteries by modulating the gut microbiome and enhancing the microbial production of SCFAs.

In conclusion, our study demonstrated a novel aspirin‐gut‐microbiota interaction that is critical for understanding the pathology of atherosclerosis. The aspirin‐resulted increase of probiotics and butyric acid prevents HFD‐induced atherosclerosis by suppressing the pyroptosis of intraplaque macrophages and preserving the gut barrier. This indicates a COX‐independent mechanism of aspirin, which may shed new insights into its clinical application. These findings also indicate the practical values of probiotics *L. murinus* and *L. johnsonii* in translational medicine. Clinical supplementation of either these probiotics or butyric acid may be a promising strategy in future prevention and therapy of ASCVD.

## 4 Experimental Section

### Human Subjects

Human fecal samples (Cohort 1) were collected from 19 healthy volunteers who received oral aspirin treatment for 30 days. A low dose of aspirin (100 mg q.d.) was used for all volunteers to determine its effects on the gut microbiome and associated metabolites. The clinical characteristics are shown in Table S1 (Supporting Information). All of the subjects enrolled satisfied the following criteria: Healthy volunteers aged 40 to 60 years old and had not been treated with aspirin in the past 1 year before this study. The baseline information of the human subjects is shown in Table S1 (Supporting Information). Human fecal samples were snap‐frozen in dry ice and stored at ‐80 °C until analysis at the Health Examination Center of the First Affiliated Hospital of Xi'an Jiaotong University. For Cohort 2, drug–microbiome associations (MetaCardis cohort) were obtained from a cross‐sectional multicenter study, which included a total of 2173 individuals (Table S2, Supporting Information).^[^ ^10b^ ^]^ Data were collected from healthy subjects and patients with ischemic heart disease. Then, the gut microbiota and metabolites affected by aspirin were reanalyzed using the MetaCardis database.

### Mice

Male *ApoE* ^−/−^ mice aged 6 to 8 weeks (body weights ≈20 g) were obtained from Beijing Vital River Laboratory Animal Technology Co., Ltd. Preliminary results indicated that atherosclerosis induced by a high‐fat diet was related to mouse gender, as male mice were more susceptible to this disease. All mice were housed in a standard specific‐pathogen‐free (SPF) environment and fed with food and water available ad libitum at the Experimental Animal Center of Xi'an Jiaotong University. The mice were individually housed controlled environment (20 ± 2 °C, 12‐h light/dark cycle).

Male *ApoE^−/−^* mice were fed with a high‐fat diet(1.25% cholesterol and 40% w/w fat) to establish an atherosclerosis model, and drinking water with vehicle or 0.2 mg mL^−1^ aspirin for 12 weeks. To determine the aspirin doses for mice, adjustments of human doses (100 mg/day/individual) were calculated, which were transformed based on body surface area. The body surface area was calculated through the Meeh‐Rubner formula: Body Surface Area m^2^ = 9:1. Non‐fasting animals were anaesthetized and sacrificed respectively. The intestinal contents and feces were collected. To clarify the role of gut microbiota in aspirin anti‐atherosclerosis treatment, classical antibiotics, 5 mg mL^−1^ of streptomycin and 0.1 mg mL^−1^ of clindamycin, were added into the drinking water of mice, leading to the elimination of gut microbiota. (Figure 2).^[^ ^6^ ^]^

For lactate intervention (Figure 5), the *ApoE* ^−/−^ mice (6‐8‐week‐old, male) were treated with HFD and lactate (100 mm in drinking water, ad libitum) for 10 weeks. Mice ingested 50 mg of sodium lactate daily through their drinking water.

For the butyric acid intervention (Figure 5), the *ApoE* ^−/−^ mice (6–8 weeks old, male) were treated with HFD and butyrate (1 mm in drinking water, ad libitum) or saline for a duration of 10 weeks.

Mice were euthanized through intravenous injection of a lethal dose of pentobarbital sodium (100 mg kg^−1^), and their organ tissues were removed and collected for further biochemical analysis.

### Bacterial Culture

The *Lactobacillus johnsonii* (ATCC33200), *Ligilactobacillus murinus* (ATCC35020), and *Allobaculum stercoricanis* (BNCC363050) strains were strewn on MRS plates or blood agar and cultured overnight at 37 °C under anaerobic conditions (10% CO_2_, 10% H_2_, and 80% N_2_) until obvious plaque appeared. A single colony was selected and strewn on plates again until obvious plaque appeared. The bacteria were collected with PBS solution and centrifuged at 300 g for 5 min. Discarded the supernatant and added PBS solution to adjust the OD600 value to 0.6 under the microplate reader, which was 1 × 10^9^ cfu mL^−1^.

### Analysis of Atherosclerotic Plaque

The heart and aortic tissues were removed from the ascending aorta to the iliac artery and then fixed in 4% paraformaldehyde. To analyze the lesion area in the aortic arch, the intimal surface was exposed by a longitudinal cut. Next, the aorta was stained in freshly prepared Oil Red O solution at room temperature and destained several times with 70% ethanol. The stained aorta was then placed on a cationic anti‐off slide and spread out completely. Images were captured using the high‐resolution camera and analyzed using ImageJ software (version 2.10), and lesion areas were assessed as the percentage of Oil Red O positive area in the surface area of the entire aorta, as shown in a previous study.^[^ ^16a^ ^]^ Hearts of AS mice were fixed in 4% paraformaldehyde, embedded in OCT compound, and sectioned (8 µm thickness) on freezing microtome Cross‐sectional frozen sections of the left ventricular outflow tracts were stained with Oil Red O, HE, and MOVAT to evaluate atherosclerotic plaque burden, necrotic core size, and fibrosis degree. Images of plaques were captured under the Olympus microscope (BX51‐FL‐CCD), and quantitative analysis was performed with ImageJ software by averaging the lesion areas in the sections.

For immunofluorescence, to detect the expression and localization of macrophages (CD68) and pyroptosis‐related proteins (NLRP3, IL‐1β) in cross sections of the left ventricular outflow tract, sections were antigen repaired with sodium citrate repair solution, broken with 0.1%Triton X‐100, and blocked with BSA. The sections were incubated overnight with antibodies CD68 (Servicebio, GB300605,1:200), NLRP3(Abcam, ab263899,1:1000), and IL‐1β (Abcam, ab283818,1:3000). HRP‐conjugated secondary antibody (Cell Signaling, 7076S, 7074S; 1:1000) and TSA kit (Servicebio) were incubated at room temperature. Sections were stained with DAPI and then scanned by fluorescence microscopy. Images of positive areas of CD68, NLRP3, and IL‐1β were captured under the Nikon Eclipse C1 microscope (Nikon, Japan), and quantitative analysis was performed with CaseViewer software by averaging the lesion areas in the sections.

### Fecal Microbiota Transplantation (FMT)

For FMT experiments in Figure 2, donor mice (8‐week‐old, male, n = 8) were treated with saline (Saline group) or aspirin (Asp group) for more than 2 weeks. Fecal samples (above 150 mg) of each mouse were collected from day 14 to day 20 and stored in sterile tubes, and homogenized in 1 mL of PBS. After centrifugation (2000 g for 10 min), bacteria‐enriched supernatants were collected and centrifuged (5 min at 15 000 g). Bacterial pellets were washed twice with phosphate buffered saline (PBS), resuspended in 700 µL of saline with 20% (v/v) glycerol, and stored at −80 °C. For recipient mice, saline‐ or HFD‐fed mice were treated with fecal microbiota transplants from each donor group via oral gavage (200 µL, 3 times/week) for 12 weeks.

### Colonization of L. Murinus and L. Johnsonii

For gavage of *L. johnsonii*, *L. murinus*, the *ApoE^−/−^* mice (6‐8‐week‐old, male) were fed with HFD for 4 weeks, then oral administration of saline or bacteria was conducted in the fifth week by gavage 3 times a week for 12 weeks (Figure 3). *ApoE^−/−^* mice were fed HFD and received oral administration of either saline or *A. stercoricanis* for a duration of 10 weeks (Figure 5). The concentration of bacteria was 1 × 10^9^ cfu mL^−1^, and ApoE^−/−^ mice were given 200 µL intra gavage.

### Cell Cultures and Reagents

Mouse monocyte‐macrophage cell line RAW264.7 was obtained from Procell Life Science & Technology with correct STR identification (CL‐0190, Procell, Wuhan). For peritoneal macrophages, 1 mL of 3% nutrient broth (247110, BD technology) was injected into the peritoneal cavity of male C57 mice for 3 days. Peritoneal fluid was acquired on the fourth day, and PMs were then isolated and cultured in DMEM (high glucose, Hyclone) supplemented with 10% FBS (Gibco, USA) in 5% CO2 incubator at 37 °C. Cells were stimulated with 100 ng mL^−1^ LPS for 24 h to construct a macrophage inflammation model in vitro. Meanwhile, butyric sodium (1 mm), Trichostatin A (10 nm), AR420626 (25 µm), 4‐CMTB (100 µm), and niacin (1 mm) were added into the medium for 24 h, accompanied by LPS stimulation. The reagents used and their commercial sources were as follows: DMSO (D8418, Sigma‐Aldrich), LPS (SMB00610, Sigma–Aldrich), Butyric sodium (567430, Sigma–Aldrich), Trichostatin A (HY‐15144, MCE), AR420626 (HY‐116522, MCE), 4‐CMTB (HY‐P1125, MCE), and Niacin (HY‐B0143, MCE).

### siRNA‐mediated Gene Knockdown

RAW264.7 macrophages were seeded into a plate and cultured to 80% confluence. siRNAs targeting GPR41, GPR43, GPR109A, and a negative control siRNA were purchased and stored following the instructions of the manufacturer (GenePharma, Shanghai, China). Cells were transfected with siRNA or negative control siRNA (50 nm) using Lipofectamine 2000 Reagent (#11668019, Thermo Fisher Scientific) following the manufacturer's protocol.

### Treatment of Aspirin on Bacteria

For the treatment of aspirin to bacteria in vitro in Figure 5, the relative concentration of live bacteria (*L. johnsonii*, *L. murinus*, and *A. stercoricanis)* was 1 × 10^9^ cfu mL^−1^. Acetylsalicylic acid (A800349, Shanghai Macklin Biochemical Technology) was dissolved in culture medium with final concentrations ranging from 0 to 0.8 mm. The OD 600 value was examined 72 h after aspirin was added.

### Serum Lipids Measurement

In Figure S1 (Supporting Information), fast serum was obtained and collected overnight from *ApoE^−/‐^* mice in different groups. Lipid levels (triglyceride, total cholesterol, low‐density lipoprotein, and high‐density lipoprotein) were detected by the automatic biochemistry analyzer Labpspect008a using the methods of ELISA in the department of clinical laboratory of the First Affiliated Hospital of Xi'an Jiaotong University.

### TMA‐TMAO Measurement

Serum samples from *ApoE^−/−^* mice stored in ‐80 °C until analysis, TMA‐TMAO‐associated metabolites levels (TMAO, TMA, Choline, L‐Carnitine, and Betaine) were measured by ultra‐high performance liquid chromatography (UHPLC) coupled with a quadrupole time‐of‐flight (QTOF). A triple quad with a 100X dynamic range was utilized for precise quantification of these metabolites using authentic standards (standard curves). The levels of metabolites in fecal samples were compared and analyzed between the four groups.

### Western Blotting

Cells were lysed in RIPA buffer supplemented with protease inhibitors (Thermo Scientific). The lysates were separated by SDS‐PAGE and immunoblotted with antibodies as indicated. The antibodies used included anti‐GAPDH (Beyotime, AF2819; 1:3000), anti‐NLRP3 (Abcam, ab263899; 1:1000), anti‐Caspase‐1 (Cell Signaling, 24232S; 1:1000), anti‐IL‐1β (Abcam, ab283818; 1:1000), anti‐GSDMD (Proteintech, Cat. No. 66387‐1‐LG; 1:1000), anti‐ZO‐1 (Servicebio, GB115686; 1:1000), and anti‐MUC2 (Abcam, ab272692; 1:1000). The secondary antibodies were anti‐mouse (Cell Signaling, 7076S; 1:1000) and anti‐rabbit (Cell Signaling, 7074S; 1:1000).

### RT‐PCR

Total RNA was extracted by using TRIzol reagent (Invitrogen) and fast 200 Kit (Fastagen). Purity of RNA was evaluated spectrophotometrically by 260/280 ratio. Reverse transcription was accomplished using PrimeScript RT Master Mix (Takara, RR036A) and 1000 ng RNA. Real‐time PCR was accomplished using TB Green Premix Ex Taq II (Takara, RR820A). The mRNA level was measured by using SYBR Green (Bio‐Rad) with GAPDH as internal controls, and all cDNA samples were assayed in duplicate. Primer sequences used for qPCR are in Table S3 (Supporting Information).

### Flow Cytometry

Cell suspension was centrifuged at 500 g for 5 min, and then resuspended and blown evenly. PE‐CD86 (Sino Biological, 50068‐R313,1:200) and APC‐CD206 (Biolegend, 141707,1:200) antibodies were added to the cell suspension and incubated for 15 min at room temperature in the experimental group, and negative control and single positive control were set up meanwhile. After resuspended and washed with flow buffer, the cell suspension was examined by a Beckman DxFLEX flow cytometer.

### Immunofluorescence

RAW264.7 cells were seeded, cultured, and intervened in confocal dishes. The cell membrane was broken by 0.1% Trition X and non‐specific binding was blocked by BSA solution. The cells were incubated with primary antibodies (NLRP3, Abcam, ab263899, 1:200; IL‐1β, Abcam, ab283818, 1:200) at 4 °C overnight. The secondary antibodies were incubated the next day, and the nucleus was stained with DAPI solution (Invitrogen, R37606, 1 µg mL^−1^), washed with PBS solution, and observed and photographed under an EVOS microscope.

### Alcian Blue Periodic acid Schiff Stain (AB‐PAS)

Intestinal sections were dewaxed in distilled water and rinsed, and then stained with alcian blue staining solution and treated with oxidant. After soaking in Schiff Reagent and dehydrated by a series of ethanol, transparented by xylene, and sealed with retinene. Finally, the digital sections were prepared using a slice scanner (Panoramic MIDI).Chiu's score: The scores were independently evaluated by two independent pathologists, and the standards were described previously.^[^ ^6^ ^]^ The mean scores were calculated based on the average values of 10 randomly selected images from a single slice of intestinal tissue, and all individuals in each group were analyzed statistically.

### Transmission Electron Microscopy (TEM)

Cells were centrifuged at 1000 rpm for 5 min, and the supernatant was discarded. Added diluted fixative (G1102, Servicebio, Wuhan, China) slowly to the wall at a ratio of 1:5 (3% glutaraldehyde: 0.1 mol L^−1^ PBS buffer). The cells were resuspended and centrifuged at 12 000 rpm for 10 min. Abandoned the supernatant and added 3% glutaraldehyde fixative slowly along the tube wall, then stored at 4 °C. Intestinal tissues of mice were fixed using electron microscopy fixative. The samples were sent to the electron microscopy laboratory of Xi'an Jiaotong University, embedded in resin, and ultrathin sections (70 nm) were cut and stained. Cell and tissue morphology was observed and analyzed by Hitachi HT‐HT7700 TEM transmission electron microscopy.

### 16S rRNA Gene Sequencing

Fecal samples from *ApoE^−/^* ^−^ mice and humans were collected for 16S rRNA sequencing (Biomarker, Beijing, China). Sequenced data were investigated by single‐molecule real‐time (SMRT) sequencing on PacBio platforms, generating circular consensus sequences (CCS) after amplification and purification of the V1‐V9 region of bacterial 16s rRNA genes. Usearch was applied to cluster reads with similarity above 97.0%, generating OTUs. Statistics on composition in each sample were calculated at the level of phylum, class, order, family, genus, and species. Alpha and Beta diversity of each sample were analyzed by QIIME2 software. ANOVA analysis was used to test the significance of the difference in gut microbiota species. The correlation analysis of gut microbiota was performed by the Pearson test. For all statistical results, a P value of less than 0.05 was considered to indicate statistical significance.

### Untargeted Metabolomics and Targeted Metabolomics Analysis

Quantitation of fecal metabolites was performed and analyzed by untargeted metabolomics (Biomarker, Beijing, China). Untargeted metabolomics was performed by LC‐MS‐based metabolomics, which was ideal for identifying and quantifying small‐molecule metabolites (<650 Da), including small acids, alcohols, hydroxyl acids, amino acids, sugars, fatty acids, sterols, catecholamines, drugs, and toxins. The SCFAs spectrum in the study samples was detected utilizing GC‐MS/MS. The raw data files exported by GC‐MS/MS were qualitatively and quantitatively processed, and actual concentrations were analyzed based on the standard samples by MWDB (Metware Database) database (Quantitative curves).

### The α‐Diversity, β‐Diversity, and Network Visualization

The α‐diversity analysis was performed using the usearch software as described^[^ ^6^ ^]^ while β‐diversity analysis was performed using the “vegan” package in R v 3.6.1 to assess the feature richness in mice feces. The α‐diversity indexes (Shannon, Simpson, Chao1, ACE, and PD whole indexes) were calculated by usearch based on the relative abundance of each taxon. β‐Diversity analysis was conducted using principal component analysis (PCA) or PCoA based on Bray Curtis dissimilarity index matrices. The networks of species‐species or genus‐genus correlations were visualized by ChiPlot (https://www.chiplot.online/). The LefSE plot and LDA score were generated through the online platform Wekemo for multi‐omics data analysis.^[^ ^44^ ^]^ The Mantel tests were conducted and visualized in R v3.6.1 using the “ggcor” package.

### Metagenomic Sequencing

For metagenomic sequencing data in Figure 4 and Figure S4 (Supporting Information), stool samples were collected as previously described from volunteers before and after aspirin treatment.^[^ ^6^ ^]^ DNA, 700 ng per sample, was used for sample preparation, and Sequencing libraries were established by NEB Next Ultra DNA Library Prep Kit for Illumina (# E7370L, NEB, USA); the manufacturer's recommendations and index codes were adopted to attribute sequences to each sample. The fragmented DNA ends were repaired, polyA‐tailed, and ligated with a sequencing adaptor for Illumina sequencing. PCR amplification and purification (AMPure XP system) were performed. The insert size of the library was assessed by using the Agilent Bioanalyzer 2100 system. The clustering of the index‐coded samples was performed on a cBot Cluster Generation System using HiSeq 4000 PE Cluster Kit (Illumina) according to the manufacturer's instructions. After cluster generation, the library preparations were sequenced on an Illumina HiSeq 4000 platform, and 150‐bp paired‐end reads were generated. For Cohort 2, the metagenomic data were retrieved from the published dataset.^[^ ^45^ ^]^

### RNA‐Seq Analysis

Mice peritoneal macrophages were isolated from male C57 mice (n = 6) and then treated with butyric acid (1 mm) for 24 h, followed by LPS stimulation. After treatment, total RNAs were isolated from macrophages with Trizol reagent (cat number: 15596026, Invitrogen, Carlsbad, CA, USA), and the sequencing libraries were prepared. Poly‐A‐containing mRNA was isolated from the total RNA by poly‐T oligo‐attached magnetic beads and then fragmented by an RNA fragmentation kit. The cDNA was synthesized using random primers through reverse transcription. After the ligation with the adaptor, the cDNA was amplified by 15 cycles of PCR, and then 200‐bp fragments were isolated using gel electrophoresis. The products were sequenced by an Illumina HiSeq2500 instrument in Shanghai Sangon Biotech Technology (Shanghai, China). The data were analyzed on the free online platform of Majorbio Cloud Platform (www.majorbio.com). The differential expression of genes was selected by a fold change of >2 (Log2FC > 1 or Log2FC < −1) and *P* adjust value<0.05.

### Single‐Cell RNA Sequencing (scRNA‐Seq)

The RNA‐seq data was from Gene Expression Omnibus (GEO) (GSE159677) produced by Alsaigh et al, containing atherosclerotic core plaques (plaque group) and ctrl group of patients. Subsequently, clustering revealed seven distinct cell populations by Uniform manifold approximation and projection (UMAP) distribution. The expression data of GPR41, GPR43, GPR109A, together with the expression of pyroptosis markers in macrophages (myeloid cells) were extracted and compared.

### Quantification and Statistical Analysis

Data in each group was analyzed by Graphpad Prism 9.0 and SPSS 23.0 software. All results were presented as mean ± S.d., except for special prompts. Before data analysis, normality was first tested using the Shapiro‐Wilk test (*P*<0.05), and homogeneity of variances was tested using the *F* test (*P*<0.05). The independent sample two‐sided Student *t*‐test or one‐way ANOVA was used to test whether the data conformed to the normal distribution, while the Kruskal‐Wallis h or Mann‐Whitney u test was used for the data not conforming to the normal distribution. For all statistical results, a *P* value of less than 0.05 was considered to indicate statistical significance. In addition, all biological experiments were repeated at least 3 times.

The cohort study was performed in accordance with the medical ethics committee of the First Affiliated Hospital of Xi'an Jiaotong University, and all patients provided informed consent. All animal procedures were approved by the Xi'an Jiaotong University Institutional Animal Care and Use Committee.

### Ethics Statement

The use of human samples complies with the Declaration of Helsinki and was approved by the Ethics Committee of Xi'an Jiaotong University (Ethical approval number: 2020–299) (Xi;an, Shaanxi, China). All the participants provided their written informed consent. All animal procedures conform to the guidelines from Directive 2010/63/EU of the European Parliament on the protection of animals used for scientific purposes or the current NIH guidelines. The animal experiments were conducted in accordance with the Guide for the Care and Use of Laboratory Animals and approved by the Institutional Animal Care and Use Committee (IACUC) at Xi'an Jiaotong University (Ethical approval number: 2021‐1373). Strain culture experiments were performed following a protocol approved by the Xi'an Jiaotong University Administrative Panel on Biosafety.

## Conflict of Interest

The authors declare no conflict of interest.

## Author Contributions

R.H. and N. D. contributed equally to this work. T.L., Z.Y., and J.L. initiated this study; T.L. conceived and supervised the whole project. R.H., N.D. performed data collection and interpretation. Y.H. and X.W. performed scRNA data collection and analysis. R.H. and N.D. guided all animal experiments, and Y. X. and X.Q. provided technical support. J.Z. and Y.W. provided statistical advice. T.L., R.H., N.D., and Y.H. prepared the figures and wrote the initial manuscript. T.B., Y.X., X.Z., and X.S. provided important discussions. T.L., Z.Y., Y.W., and J.L. provided funding acquisition. All authors approved the final manuscript.

## Supporting information



Supporting Information

## Data Availability

Research data are not shared.
