# Supplementary material for: Ligilactobacillus Murinus and Lactobacillus Johnsonii Suppress Macrophage Pyroptosis in Atherosclerosis through Butyrate‐GPR109A‐GSDMD Axis
Source: Adv Sci (Weinh). 2025 Jul 29;12(38):e01707. doi: 10.1002/advs.202501707 (PMC12520491; doi:10.1002/advs.202501707)
Supplement: Supplementary file 1 — Supporting Information [file ADVS-12-e01707-s001.pdf]

ADVANCED SCIENCE
Open Access

Supporting Information

for *Adv. Sci.*, DOI 10.1002/advs.202501707

Ligilactobacillus Murinus and Lactobacillus Johnsonii Suppress Macrophage Pyroptosis in Atherosclerosis through Butyrate-GPR109A-GSDMD Axis

Rui Hua, Ning Ding, Yiming Hua, Xiaoke Wang, Yu Xu, Xiangrui Qiao, Xue Shi, Ting Bai, Ying Xiong, Xiaozhen Zhuo, Chong Fan, Juan Zhou, Yue Wu, Junhui Liu, Zuyi Yuan* and Ting Li**

Ligilactobacillus murinus **and** ***Lactobacillus johnsonii*** **Suppress Macrophage Pyroptosis in Atherosclerosis Through Butyrate-GPR109A-GSDMD Axis**

Rui Hua, Ning Ding, Yiming Hua, Xiaoke Wang, Yu Xu, Xiangrui Qiao, Xue Shi, Ting Bai, Ying Xiong, Xiaozhen Zhuo, Chong Fan, Juan Zhou, Yue Wu, Junhui Liu, Zuyi Yuan and Ting Li

Supplementary information includes:

Supplementary Tables:

Table S1. Demographic characteristics of the subjects in the Cohort 1.

Table S2. Demographic characteristics of the subjects in Cohort 2.

Table S3. Sequence of PCR primers and siRNAs.

Supplementary Figures:

Figure S1 Aspirin reshapes gut microbiome without affecting serum lipids and TMAO features.

Figure S2 FMT increases probiotics and inhibits progression of atherosclerosis.

Figure S3 *L. murinus* and *L. johnsonii* shift gut microbiome towards probiotics.

Figure S4 Aspirin increases gut butyric acid in human cohorts.

Figure S5 Expression of GPR41 and GPR43 in intraplaque macrophages.

Figure S6 Schematic model of gut microbiota-butyrate-GPR109A-GSDMD Axis.

Table S1. Demographic characteristics of the subjects in the Cohort 1

Characteristics (N=19)		Before aspirin
		Mean (SD)
Basic information	Gender (Female %)	8(42.11)
	Age (years)	46.58 (12.41)
	SBP (mmHg)	123.74 (18.68)
	DBP (mmHg)	79.37 (9.61)
	Hypertension (%)	4 (21.05)
	T2DM (%)	2 (10.53)
	Aspirin (%)	0(0)
	Statin (%)	1 (5.26)
	β-blocker (%)	5 (26.32)
	ACEI/ARB (%)	1 (5.26)
	CCB (%)	2(10.53)
Routine blood test	WBC (10^9/L)	6.35 (1.54)
	Hb (g/L)	142.58(16.18)
	Platelet (10^{12}/L)	207.05(60.34)
	Hs-CRP	3.59(7.98)
Renal function	Scr (μmol/L)	49.93 (15.04)
	BUN (mmol/L)	9.89(16.79)
Serum lipid	LDLc (mmol/L)	2.10 (0.84)
	TG (mmol/L)	1.73 (0.59)
	HDLc (mmol/L)	0.97(0.22)
	TC (mmol/L)	3.68 (0.87)
Serum glucose	HbA1c (%)	5.99(0.84)

Abbreviations: ACEI: Angiotensin converting enzyme inhibitors; ARB: Angiotensin receptor blocker; BUN: Blood urea nitrogen; CCB: Calcium channel blocker; DBP: Diastolic blood pressure; Hb: haemoglobin; HbA1c: Haemoglobin A1C; HDLc: High-density lipoprotein cholesterol; hs-CRP: high-sensitive-C-reactive protein; LDLc: Low-density lipoprotein cholesterol; Scr: Serum creatinine; TG: Triglyceride; TC: Total cholesterol; WBC: White blood cell number.

Table S2. Demographic characteristics of the subjects in Cohort 2

Characteristics		HC (N=275)		IHD-Nonuser (N= 83)		IHD-User (N=289)		
		Mean/ Incidence	SD	Mean/ Incidence	SD	Mean/ Incidence	SD	$P^{\#}$
Basic information	Sex (Female %)	62.18	N/A	12.9	N/A	17.71	N/A	<0.0001
	Age (years)	55.45	12.70	60.64	9.88	61.70	8.26	<0.0001
	SBP (mmHg)	123.32	15.94	129.25	17.93	125.76	15.17	0.038
	DBP (mmHg)	69.06	8.78	72.79	9.89	69.40	9.51	0.057
	T2DM (%)	0	N/A	27.71	N/A	31.49	N/A	<0.0001
	Physical activity(hours/week)	8.22	5.25	5.68	5.41	6,58	5.57	0.002
Medication	Aspirin (%)	1.45	N/A	0	N/A	100	N/A	<0.0001
	Statin (%)	3.64	N/A	42.17	N/A	93.08	N/A	<0.0001
	β-blocker (%)	4.73	N/A	36.14	N/A	79.24	N/A	<0.0001
	ACEI/ARB (%)	2.55	N/A	38.55	N/A	63.67	N/A	<0.0001
	CCB (%)	0.73	N/A	19.28	N/A	15.92	N/A	<0.0001
	TG (mmol/L)	0.89	0.4	1.63	0.96	1.34	0.75	<0.0001
	CRP (mmol/L)	1.78	2.92	11.78	24.46	4.44	12.42	<0.0001
	HbA1c (%)	5.42	0.27	6.48	1.36	6.15	0.98	<0.0001
	LVEF (%)	65.1	5.52	46.75	14.83	50.47	12.52	<0.0001

#: Chi-square test or Fisher's precision probability test were used for statistical analysis of categorical variable data; One-way analysis of variance was used for statistical analysis of continuous variable data.

Abbreviations: ACEI: Angiotensin converting enzyme inhibitors; ARB: Angiotensin receptor blocker; CCB: Calcium channel blocker; CRP: C-reactive protein; DBP: Diastolic blood pressure; LVEF: Left ventricular ejection fraction; SBP: systolic blood pressure; T2DM: Type 2 diabetes mellitus; TG: Triglyceride; HbA1c: Haemoglobin A1C. DBP: Diastolic blood pressure.

Table S3. Sequences of the real time PCR primers and siRNAs

	PCR primers	
TNF-α (M)	Forward Primer:	5'-TATGGCTCAGGGTCCAACTC-3'
	Reverse Primer:	5'-GGAAAGCCCATTTGAGTCCT-3'
IL-6 (M)	Forward Primer:	5'-TGTGCAATGGCAATTCTGAT-3'
	Reverse Primer:	5'-GGTACTCCAGAAGACCAGAGGA-3'
MCP-1 (M)	Forward Primer:	5'-GTCCCTGTCATGCTTCTGG-3'
	Reverse Primer:	5'-GCGTTAACTGCATCTGGCT-3'
Gapdh (M)	Forward Primer:	5’-GGGAAACTGTGGCGTGAT-3’
	Reverse Primer:	5′-CACCCTGTTGCTGTAGCCGT-3′
GPR41 (M)	Forward Primer:	5'-GTCTGTTGGTTCCTGGCATC-3'
	Reverse Primer:	5'-CCTGGCTGTAGGTTGCATTT-3'
GPR43 (M)	Forward Primer:	5'-TCCTAGACCCAGTGACTGGTGAC-3'
	Reverse Primer:	5'-TTTGTACATGTGCTCCGCTGA-3'
GPR109A (M)	Forward Primer:	5'-CTGGAGGTTCGGAGGCATC-3'
	Reverse Primer:	5'-TCGCCATTTTTGGTCATCATGT-3'
	siRNAs	
SiGPR41	Sense	5'-CCGGCCACUGUAUGGAGUGAU-3’
	Antisense	5'-AUCACUCCAUACAGUGGCCGG-3’
SiGPR43	Sense	5'-CACUGUAGUGUGGUUUACA-3’
	Antisense	5'-UGUAAACCACACUACAGUG-3’
SiGPR109A	Sense	5'-CGGACAACUAUGUCCAUAA-3’
	Antisense	5'-UUAUGGACAUAGUUGUCCG-3’
NC siRNA	Sense	5'-UUCUCCGAACGUGUCACGUTT-3’
	Antisense	5'-ACGGUACACGUUCGGAGA ATT-3’

Supplementary Figures

Figure S1

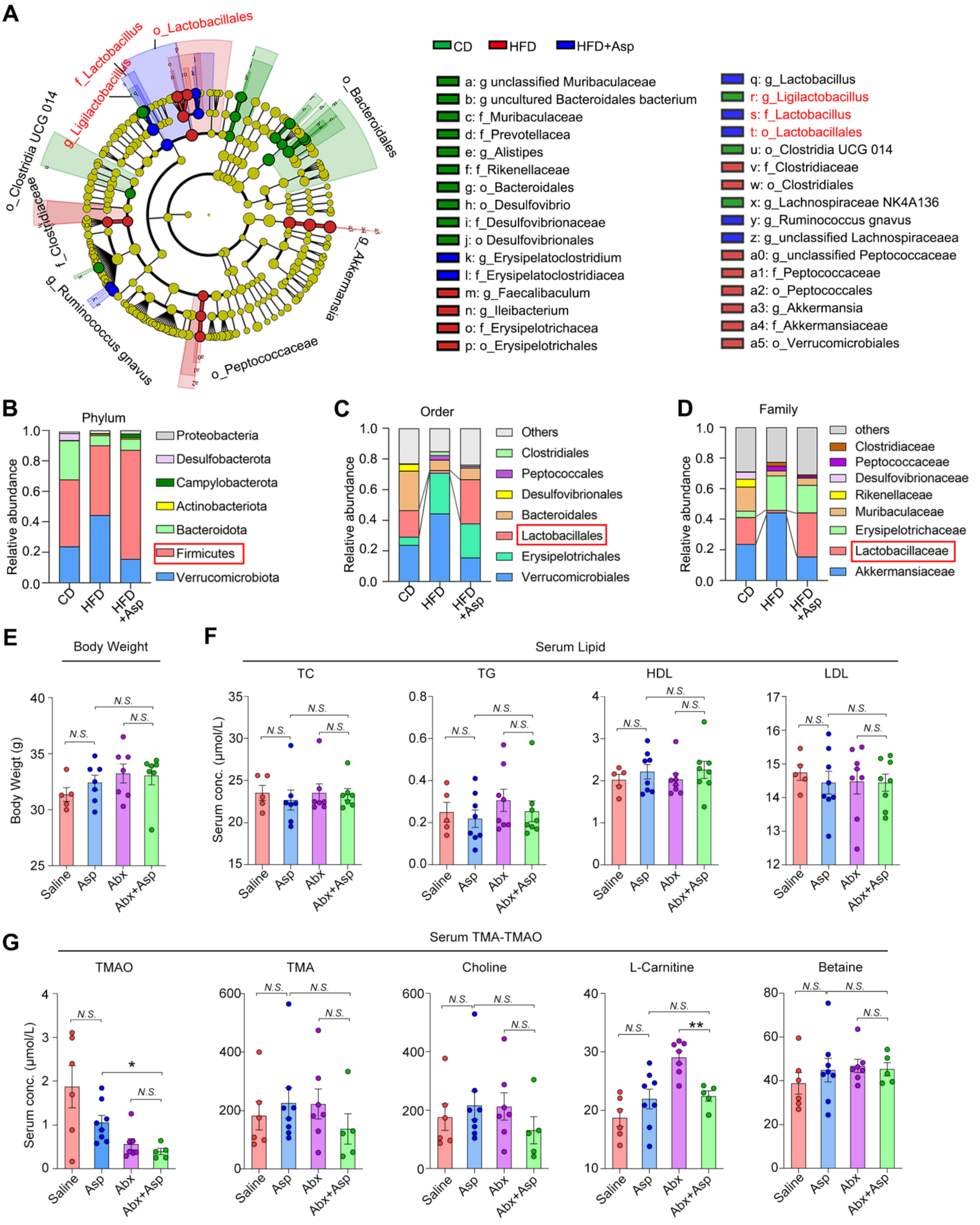

Figure S1 Aspirin reshapes gut microbiome without affecting serum lipids and TMAO features.

(A) Linear discriminant analysis (LDA) score in the CD- and HFD-fed *ApoE*$^{-/-}$ mice were treated with low-dose aspirin as described in Figure 1A.

(B-D) Changes of relative abundance of gut microbiota at levels of phylum (B), order (C), family (D) among the three groups.

(E) The body weight of *ApoE*$^{-/-}$ mice treated as in Figure 1A. The body weight data were collected before the mice were sacrificed. n=5-7/group.

(F) Serum lipids including total cholesterol (TC), triglyceride (TG), high-density lipoprotein-cholesterol (HDL) and low-density lipoprotein-cholesterol (LDL) were detected by ELISA before the mice were sacrificed. n=5-8/group.

(G) Serum TMAO and related metabolites were detected by liquid chromatograph mass spectrometer (LC-MS) before the mice were sacrificed. TMAO, trimethylamine oxide; TMA, trimethylamine. n=5-7/group.

Data are expressed as mean ± s.d. One-way ANOVA followed by Fisher's LSD post hoc test (E-G).

Figure S2

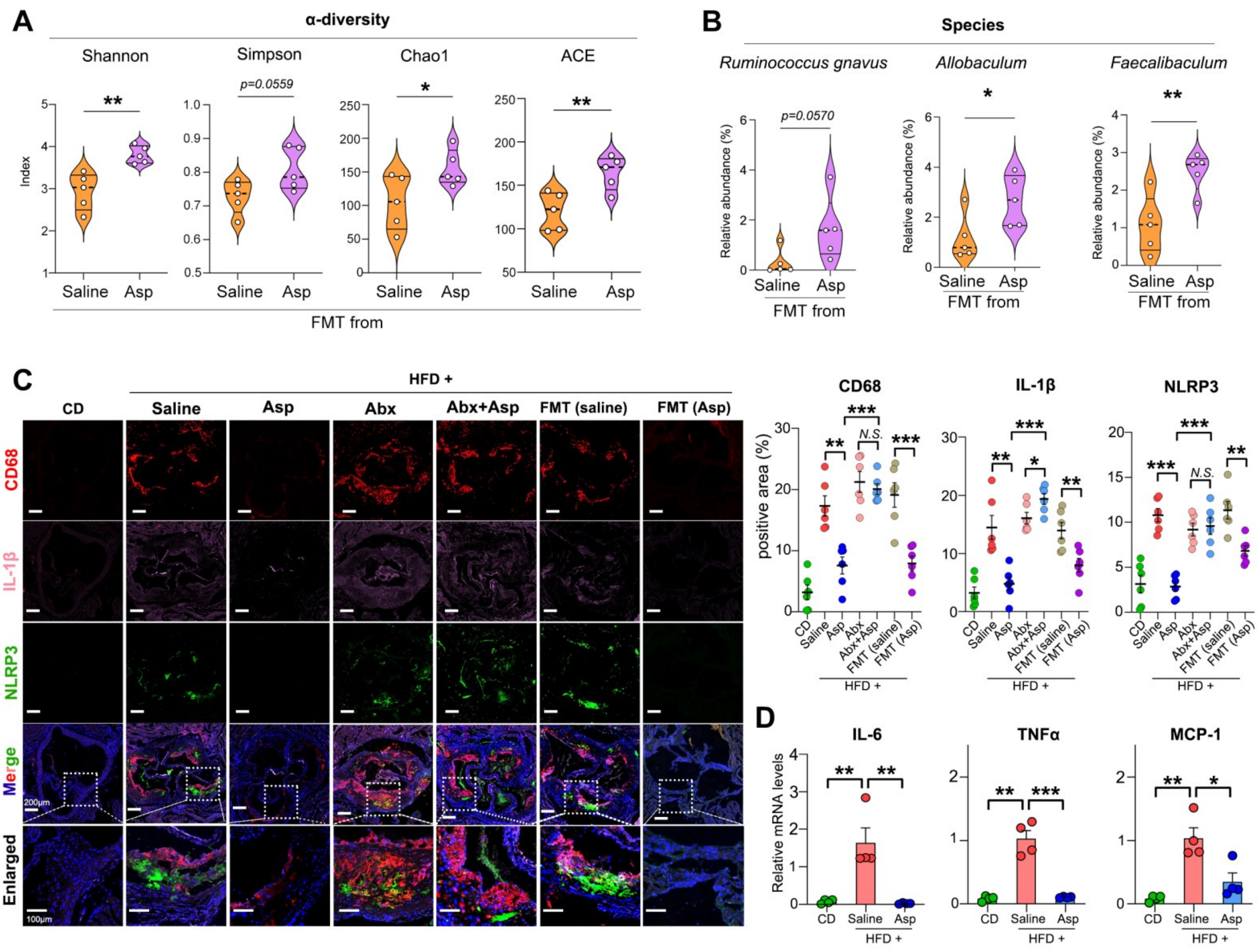

Figure S2 FMT increases probiotics and inhibits progression of atherosclerosis.

(A) The α-diversity of gut microbiome in fecal samples of recipient mice after FMT from donor mice treated with aspirin, as described in Figure 2E. n=5/group.

(B) Relative abundance of *Ruminococcus gnavus*, *Allobaculum* and *Faecalibaculum* after FMT from control and asp-treated mice. n=5/group.

(C) Representative images (left) of immunofluorescence staining for CD68 (red), NLRP3 (green), and IL-1β (pink) in the left ventricular outflow tracts, and the positive areas were statistically analysed (right). Scale bar: Upper, 200 μm; bottom: 100 μm. n=5/group.

(D) The relative mRNA expression levels of the inflammatory factors including TNF-α, MCP-1 and IL-6 in the aortas of AS mice treated with HFD and aspirin. GAPDH were used as inner control. n=4/group.

Data are expressed as mean ± s.d. Two-tailed wilcoxon rank-sum test (A-B). One-way ANOVA followed by Fisher's LSD post hoc test (C-D).

Figure S3

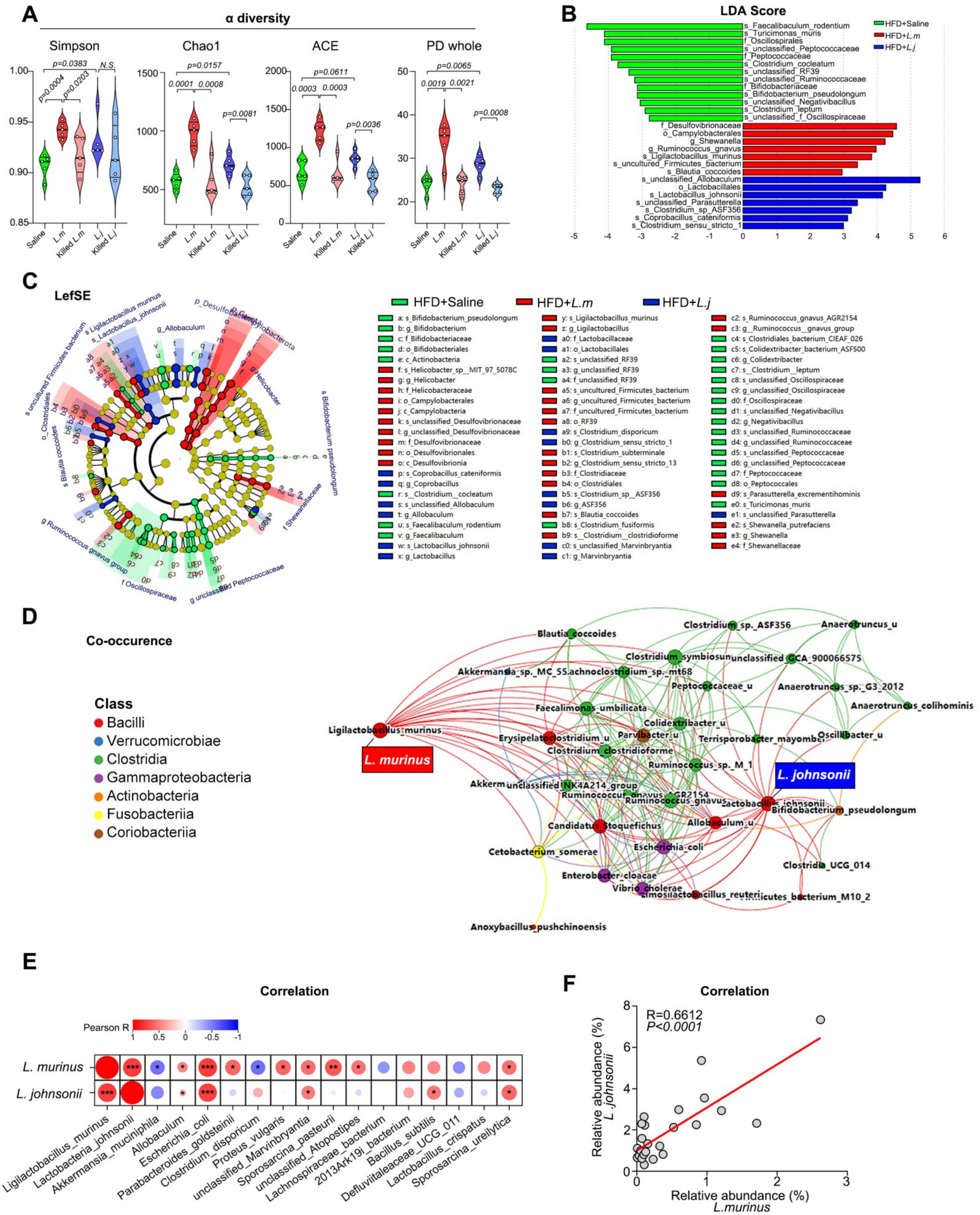

A
α diversity
Simpson
Chao1
ACE
PD whole
Saline
L.m
Killed L.m
L.j
Killed L.j
B
LDA Score
HFD+Saline
HFD+L.m
HFD+L.j
C
LefSE
D
Co-occurence
Class
Bacilli
Verrucomicrobiae
Clostridia
Gammaproteobacteria
Actinobacteria
Fusobacteriia
Coriobacteriia
L. murinus
L. johnsonii
E
Correlation
Pearson R
F
Correlation
R=0.6612
P<0.0001
Relative abundance (%) L. johnsonii
Relative abundance (%) L.murinus

Figure S3 *L. murinus* and *L. johnsonii* shift gut microbiome towards probiotics.

(A) The α-diversity of gut microbiome in fecal samples of HFD-fed mice after *L. murinus* and *L. johnsonii* transplantation, as described in Figure 3A. n=5/group.

(B) Linear discriminant analysis (LDA) scores in of HFD-fed mice treated with Saline, *L. murinus* and *L. johnsonii*.

(C) Linear discriminant analysis Effect Size (LEfSe) analysis of relative abundance of the gut microbiome across the three groups, as shown in (B).

(D) Gut microbial co-occurrence network analysis based on core genus (top 80 abundant species) in the *L. murinus* and *L. johnsonii* groups. Connecting lines indicate the absolute values of Spearman's rank correlation coefficient >0.30. Colours of nodes indicates the Class of respective species.

(E) Pearson R correlation analysis of *L. murinus* and *L. johnsonii* with other species.

(F) Correlation between relative abundance of *L. murinus* with *L. johnsonii* in stool samples in each group including HFD, HFD + Asp, HFD + *L. m* and HFD + *L. j.*

Data are expressed as mean ± s.d. ANOVA followed by Fisher's LSD post hoc test (A).
Pearson's R correlation based on relative abundance (E, F).

Figure S4

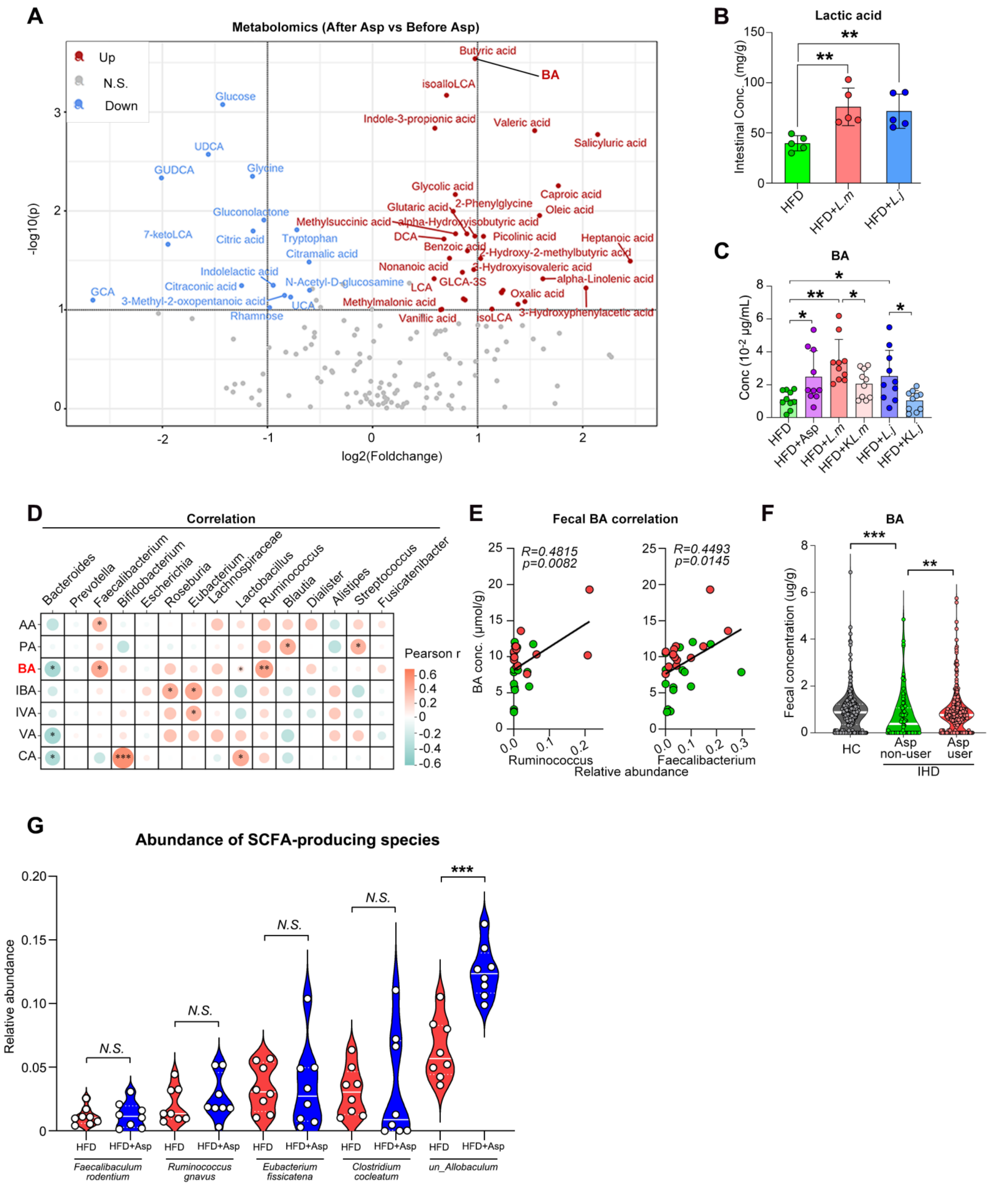

A
Metabolomics (After Asp vs Before Asp)
Up
N.S.
Down
Butyric acid
BA
isoalloLCA
Glucose
Indole-3-propionic acid
Valeric acid
Salicyluric acid
UDCA
GUDCA
Glycine
Glycolic acid
Caproic acid
2-Phenylglycine
Glutaric acid
Oleic acid
Gluconolactone
Methylsuccinic acid
alpha-Hydroxyisobutyric acid
7-ketoLCA
Citric acid
Tryptophan
DCA
Picolinic acid
Heptanoic acid
Benzoic acid
2-Hydroxy-2-methylbutyric acid
Citramalic acid
Nonanoic acid
2-Hydroxyisovaleric acid
Indolelactic acid
alpha-Linolenic acid
N-Acetyl-D-glucosamine
LCA
GLCA-3S
Citraconic acid
GCA
Oxalic acid
3-Methyl-2-oxopentanoic acid
Methylmalonic acid
UCA
Rhamnose
Vanillic acid
isoLCA
3-Hydroxyphenylacetic acid
-log10(p)
log2(Foldchange)
B
Lactic acid
Intestinal Conc. (mg/g)
HFD
HFD+L.m
HFD+L.j
C
BA
Conc (10^-2 μg/mL)
HFD
HFD+Asp
HFD+L.m
HFD+KL.m
HFD+L.j
HFD+KL.j
D
Correlation
Bacteroides
Prevotella
Faecalibacterium
Bifidobacterium
Escherichia
Roseburia
Eubacterium
Lachnospiraceae
Lactobacillus
Ruminococcus
Blautia
Dialister
Alistipes
Streptococcus
Fusicatenibacter
AA
PA
BA
IBA
IVA
VA
CA
Pearson r
E
Fecal BA correlation
R=0.4815
p=0.0082
R=0.4493
p=0.0145
BA conc. (μmol/g)
Ruminococcus
Faecalibacterium
Relative abundance
F
BA
Fecal concentration (ug/g)
HC
Asp non-user
Asp user
IHD
G
Abundance of SCFA-producing species
Relative abundance
N.S.
HFD
HFD+Asp
Faecalibaculum rodentium
Ruminococcus gnavus
Eubacterium fissicatena
Clostridium cocleatum
un_Allobaculum

Figure S4 Aspirin increases gut butyric acid in human cohorts.

(A) Volcano map of abundance of differential metabolite analysis biotarget metabolomic detection and GC/MS. Red: Metabolites with increased abundance after Asp administration. Blue: Metabolites with decreased abundance after Asp administration. Grey: Not significant.

(B) Lactate concentration measured in feces of HFD-treated *ApoE*$^{-/-}$ mice and mice transplantation of *L. johnsonii* and *L. murinus* in Figure 3A.

(C) Concentrations of butyric acid (BA) in serum samples of different groups as treated in Figure 2A and 3A.

(D) Heatmap of the correlation is shown between the abundance of species of gut microbiota and the SCFAs levels in Cohort 1.

(E) Corelations between the concentrations on fecal butyric acid and relative abundance of *Ruminococcus* and *Faecalibacterium* in Cohort 1. Green: before Aspirin; Red: after Aspirin.

(F) Relative abundance of butyric acid from fecal samples of Cohort 2.

(G) The relative abundance of the SCFAs-producing species, *Faecalibaculum rodentium*, *Ruminococcus gnavus*, *Eubacterium fissicatena*, *Clostridium cocleatum* and *un_Allobaculum* (*unclassified Allobaculum*) from AS mice treated with HFD and aspirin. n=8/group, *N.S, P*>0.05. ***, *P*<0.001.

Data are expressed as mean ± s.d. ANOVA followed by Fisher's LSD post hoc test (B, C, F). Pearson's R correlation based on relative abundance (D, E). Student t test and wilcoxon rank-sum test (G)

Figure S5

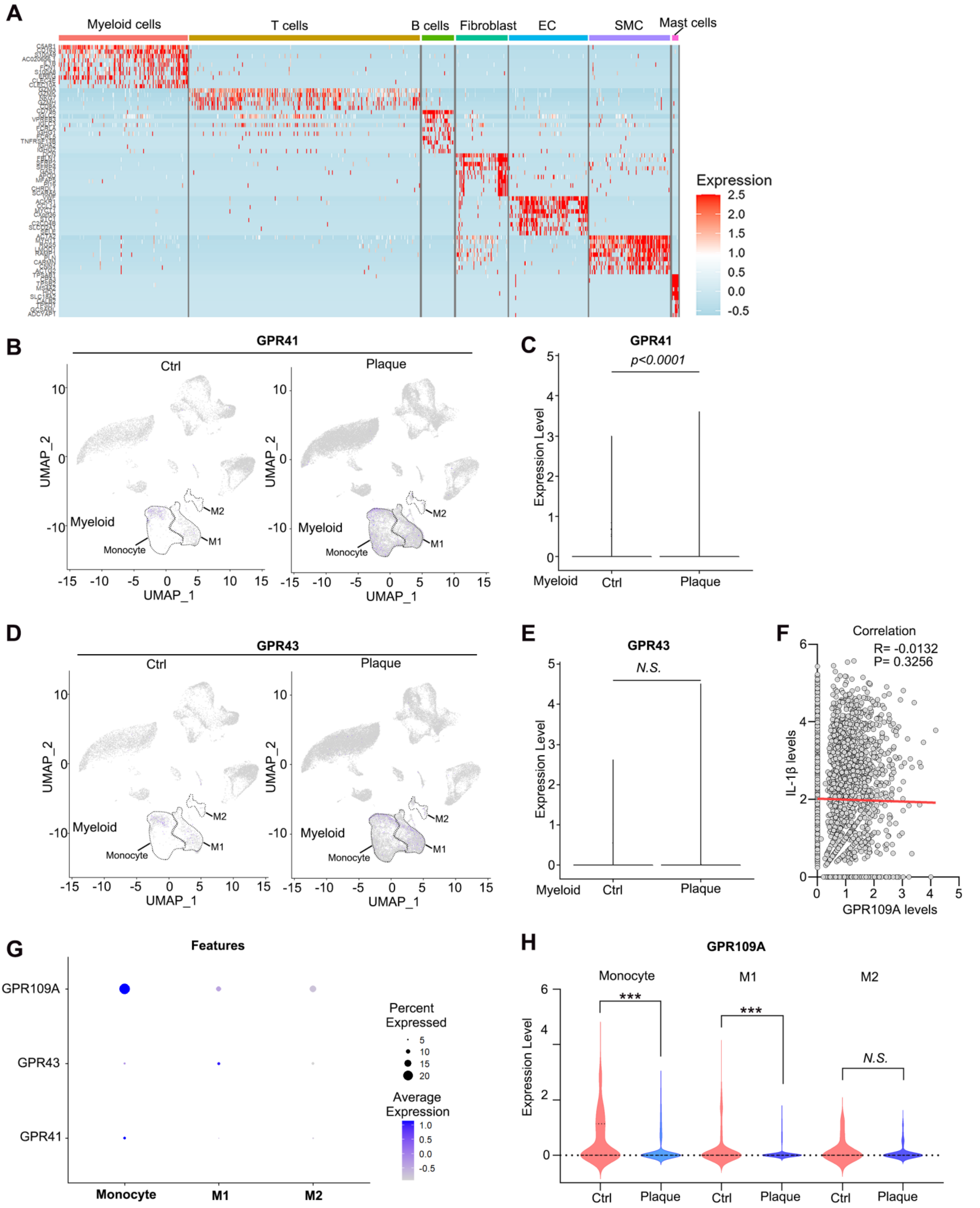
A
Myeloid cells
T cells
B cells
Fibroblast
EC
SMC
Mast cells
Expression
2.5
2.0
1.5
1.0
0.5
0.0
-0.5
B
GPR41
Ctrl
Plaque
UMAP_2
UMAP_1
Myeloid
Monocyte
M1
M2
C
GPR41
p<0.0001
Expression Level
Myeloid
Ctrl
Plaque
D
GPR43
Ctrl
Plaque
UMAP_2
UMAP_1
Myeloid
Monocyte
M1
M2
E
GPR43
N.S.
Expression Level
Myeloid
Ctrl
Plaque
F
Correlation
R= -0.0132
P= 0.3256
IL-1β levels
GPR109A levels
G
Features
GPR109A
GPR43
GPR41
Monocyte
M1
M2
Percent Expressed
5
10
15
20
Average Expression
1.0
0.5
0.0
-0.5
H
GPR109A
Monocyte
M1
M2
***
***
N.S.
Expression Level
Ctrl
Plaque
Ctrl
Plaque
Ctrl
Plaque

Figure S5 Expression of GPR41 and GPR43 in intraplaque macrophages.

(A) Expression patterns of distributions related to the UMAP's marker genes.

(B) Biaxial scatter plots showing the expression pattern of GPR41 among different subgroups of aorta cells.

(C) The differential expression of GPR41 between control and plaque groups by biaxial scatter plot and statistical analysis.

(D) Biaxial scatter plots showing the expression pattern of GPR43 among different subgroups of aorta cells.

(E) The differential expression of GPR43 between control and plaque groups by biaxial scatter plot and statistical analysis. Fisher's LSD post hoc test was used.

(F) Correlation between expressions of IL-1β and GPR109A in myeloid cells.

(G) The differential expression of GPR41, GPR43 and GPR109A in monocyte, M1 and M2 cells.

(H) The differential expression of GPR109A between control and plaque groups by biaxial scatter plot and statistical analysis.

(C, E, H) Two tailed Wilcoxon rank sum test; (F) Pearson's R correlation.

Figure S6

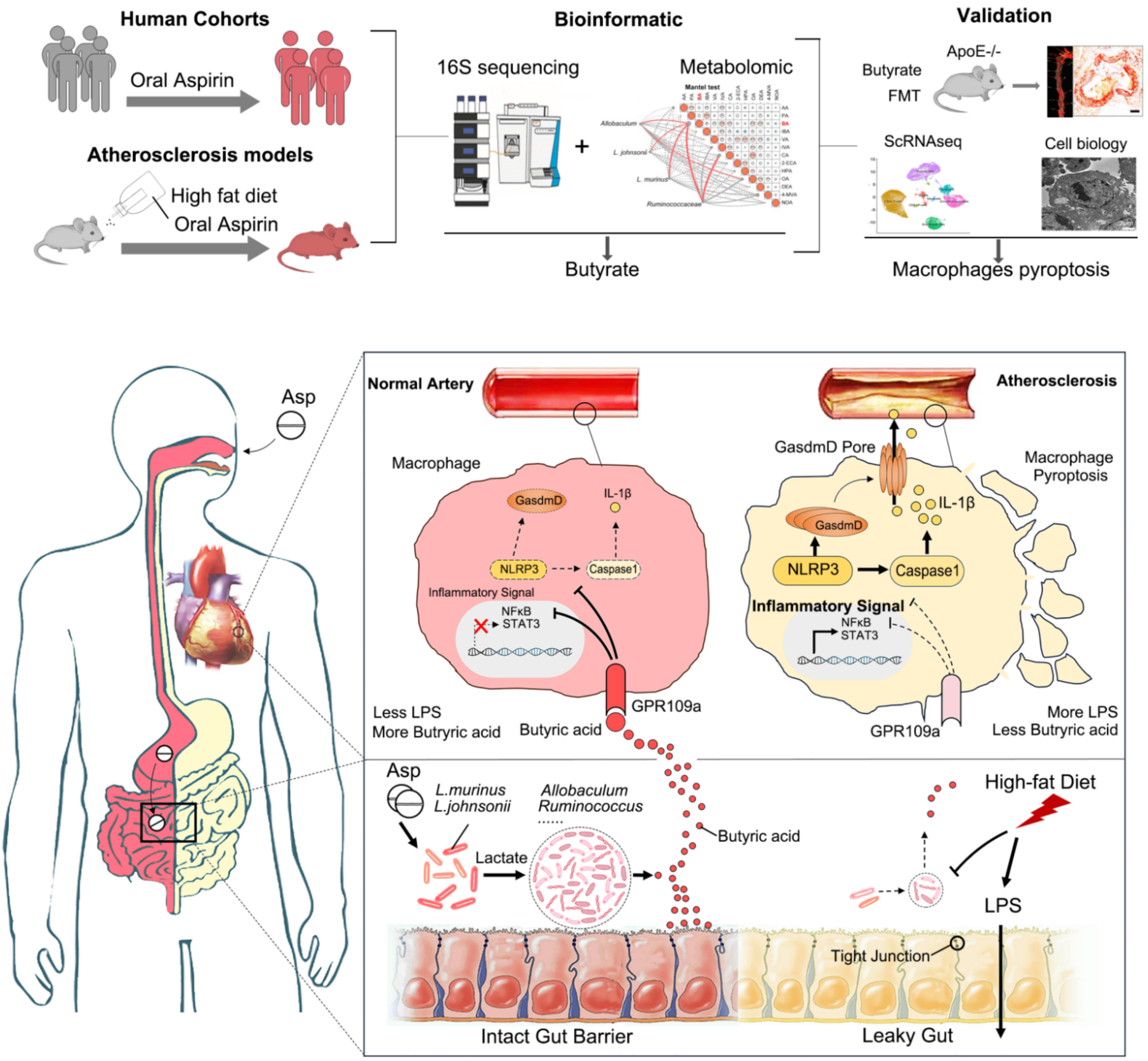

Figure S6 Schematic model of gut microbiota-butyrate-GPR109A-GSDMD Axis.

Oral low-dose aspirin lead to expansion of probiotics including *L. murinus* and *L. johnsonii*, which in turn confers the production of butyrate by providing lactate and promoting growth of butyrate-producing bacteria such as *Allobaculum*. Butyrate helps preserve the gut barrier and stop LPS from being leaked into circulation. Through binding to its receptor GPR109A on macrophage, gut microbial butyrate suppresses LPS-induced pyroptosis through GSDMD/NLRP3/IL-1β pathway.
